# C/EBPδ drives key endocrine signals in the human amnion at parturition

**DOI:** 10.1002/ctm2.416

**Published:** 2021-06-10

**Authors:** Jiang‐Wen Lu, Wang‐Sheng Wang, Qiong Zhou, Li‐Jun Ling, Hao Ying, Yun Sun, Leslie Myatt, Kang Sun

**Affiliations:** ^1^ Center for Reproductive Medicine, Ren Ji Hospital School of Medicine, Shanghai Jiao Tong University Shanghai P.R. China; ^2^ Shanghai Key Laboratory for Assisted Reproduction and Reproductive Genetics Shanghai P.R. China; ^3^ Department of Obstetrics and Gynecology Ren Ji Hospital, School of Medicine, Shanghai Jiao Tong University Shanghai P.R. China; ^4^ Shanghai First Maternity and Infant Hospital Tongji University School of Medicine Shanghai P.R. China; ^5^ Department of Obstetrics and Gynecology Oregon Health and Science University Portland Oregon USA

**Keywords:** 11β‐HSD1, C/EBPδ, COX‐2, fetal membranes, parturition

## Abstract

Amnion‐derived prostaglandin E2 (PGE2) and cortisol are key to labor onset. Identification of a common transcription factor driving the expression of both cyclooxygenase‐2 (COX‐2) and 11β‐hydroxysteroid dehydrogenase 1 (11β‐HSD1), the key enzymes in their production, may hold the key to the treatment of pre‐term labor. Here, we have found that the CCAAT enhancer binding protein δ (C/EBPδ) is such a transcription factor which underlies the feed‐forward induction of COX‐2 and 11β‐HSD1 expression by their own products PGE2 and cortisol in human amnion fibroblasts so that their production would be ensured in the amnion for the onset of labor. Moreover, the abundance of C/EBPδ in the amnion increases along with COX‐2 and 11β‐HSD1 at term and further increases at parturition. Knockout of C/EBPδ in mice delays the onset of labor further supporting the concept. In conclusion, C/EBPδ pathway may be speculated to serve as a potential pharmaceutical target in the amnion for treatment of pre‐term labor.

## Graphical abstract

– The expression of C/EBPδ increases with gestation age and in parturition in the amnion of the fetal membranes.
– C/EBPδ is capable of inducing cortisol regeneration and prostaglandin production, the key endocrine events in parturition initiation, in the amnion.
– Inhibition of the C/EBPδ pathway may delay labor onset in preterm birth.

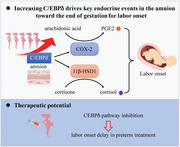

## 1 INTRODUCTION

Signals originating from the fetal membranes are pivotal in the onset of parturition.^1^, ^2^ Understanding the genesis of these signals may help develop strategies for the prevention of pre‐term birth, a leading cause of perinatal morbidity and mortality.^3^, ^4^ Substantial evidence indicates that prostaglandins (PGs) (particularly PGE2 and PGF2α) are among the most critical fetal membranes‐derived endocrine signals that are involved in the initiation and progression of parturition.^5^, ^6^, ^7^ Specifically, PGE2 and PGF2α induce cervical ripening and myometrial contractility at the onset of labor.^8^, ^9^ Their crucial roles in parturition are illustrated not only by their increased synthesis in intrauterine tissues at parturition, but also by changes in the course of parturition when their synthesis is manipulated.^10^, ^11^, ^12^

In addition to PGs, emerging evidence indicates that glucocorticoids regenerated through the reductase activity of 11β‐hydroxysteroid dehydrogenase 1 (11β‐HSD1) appear to be another important fetal membrane‐derived endocrine signal in parturition.^13^ Glucocorticoids regenerated through 11β‐HSD1 are involved not only in extracellular matrix remodeling associated with rupture of fetal membranes,^14^, ^15^, ^16^, ^17^ but also in the stimulation of PG synthesis through paradoxical induction of COX‐2 expression in amnion fibroblasts,^18^, ^19^, ^20^, ^21^, ^22^ the most significant source of PGs in the fetal membranes.^18^, ^22^, ^23^, ^24^ As a classical anti‐inflammatory hormone, glucocorticoids are well known to exert potent inhibition rather than induction of COX‐2 expression via inhibition of the nuclear factor kappa‐light‐chain‐enhancer of activated B cells (NF‐κB) in most other cell types.^25^, ^26^ Surprisingly, this induction of COX‐2 expression by glucocorticoids in amnion fibroblasts can be achieved in the presence of inhibition of NF‐κB, suggesting that a unique transcription mechanism operates in the regulation of COX‐2 expression in amnion fibroblasts. Our previous studies have revealed that this unique mechanism involves activation of the transcription factors CREB and STAT3.^19^, ^27^, ^28^ Of interest, in addition to COX‐2, glucocorticoids also induce the expression of its own regenerating enzyme 11β‐HSD1 in amnion fibroblasts so that a feedforward reaction for glucocorticoid regeneration and PG production is ensured toward the end of gestation in the fetal membranes in preparation for the onset of parturition.^29^, ^30^ Apart from the induction of 11β‐HSD1 and COX‐2 expression by glucocorticoids,^31^, ^32^, ^33^, ^34^ a number of other factors pertinent to parturition such as PGs and proinflammatory cytokines have also been shown to induce the expression of both 11β‐HSD1 and COX‐2 in amnion fibroblasts,^29^, ^35^, ^36^, ^37^, ^38^ which further illustrates the crucial roles of these genes in parturition.

Given the crucial feedforward reactions catalyzed by 11β‐HSD1 and COX‐2 in parturition, we are interested in revealing a possible universal transcription factor that may simultaneously mediate the induction of 11β‐HSD1 and COX‐2 expression by factors known to be involved in the onset of labor so that a potential therapeutic drug target can be defined for the simultaneous control of both glucocorticoid and PG production in the treatment of pre‐term labor. In order to identify this transcription factor, we performed RNA‐sequencing (RNA‐seq) in amnion fibroblasts upon cortisol treatment and in amnion tissue following labor. We found that CCAAT/enhancer binding protein delta (*CEBPD*), the gene encoding CCAAT enhancer‐binding protein δ (C/EBPδ), was among the top transcription factors that were upregulated by both cortisol and labor. C/EBPδ is a member of the CEBP family which comprises six members of basic leucine zipper transcription factors known as C/EBPα, β, δ, γ, ε, and ζ.^39^ *In silico* analysis shows that the core promoter regions of both *HSD11B1* and *PTGS2*, the genes encoding 11β‐HSD1 and COX‐2, respectively, harbor the binding sites for C/EBPs,^40^, ^41^ and the expression of both 11β‐HSD1 and COX‐2 has been shown to be subject to the regulation by C/EBP family members in a number of cell types.^31^, ^32^, ^33^, ^34^, ^41^, ^42^, ^43^

**Significance Statement**

Pre‐term birth is the leading cause of perinatal death. However, there are no effective therapeutic approaches to prevent it. We show here that C/EBPδ, a transcription factor driving gene expression, is capable of inducing key endocrine events in the fetal membranes in labor onset. Therefore, the C/EBPδ pathway may serve as a novel pharmaceutical target for treatment of pre‐term labor.

In order to investigate whether C/EBPδ is the transcription factor driving both 11β‐HSD1 and COX‐2 expression under the control of known factors involved in the onset of labor in the amnion, we first examined gestational and labor‐induced changes of C/EBPδ in human amnion. Then, we examined the role of C/EBPδ in the regulation of 11β‐HSD1 and COX‐2 expression by known factors involved in parturition in primary amnion fibroblasts. Finally, we investigated whether manipulation of C/EBPδ expression can indeed change the course of parturition by using *Cebpd* null mice.

## 2 RESULTS

### 2.1 Identification of the core transcription factor in amnion tissue and fibroblasts with the transcriptome study

**FIGURE 1:**
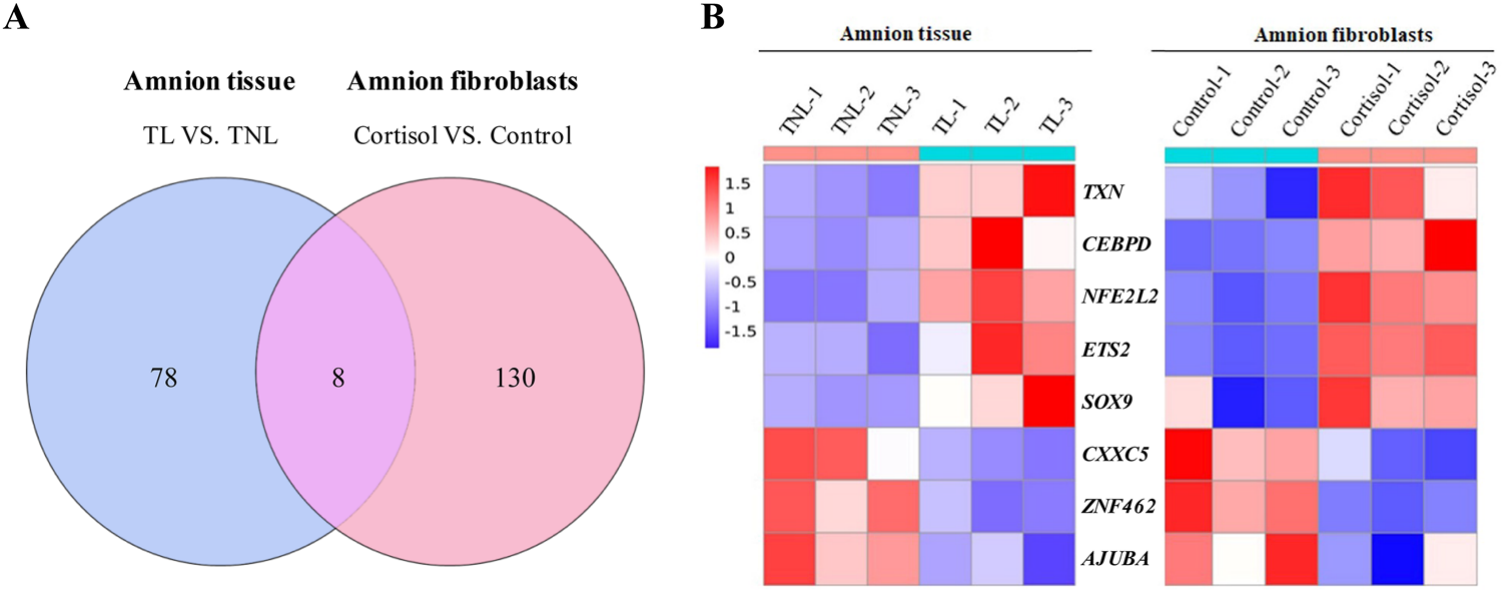
Transcription factors regulated by both labor in human amnion tissue and cortisol treatment in amnion fibroblasts. (A) Venn diagram showing overlap in transcription factors (>1.5‐fold changes) that were regulated both by labor in the amnion tissue and by cortisol treatment (1 μM; 24 h) in amnion fibroblasts. Blue circle indicates transcription factors (86 genes) which changed at parturition in the amnion tissue; red circle indicates transcription factors (138 genes) which were regulated by cortisol in amnion fibroblasts. (B) Heatmap of the transcription factors that were regulated by both labor and cortisol. Left six amnion tissue samples obtained from term pregnancies without labor (TNL) (*n* = 3) and with labor (TL) (*n* = 3). Right, paired amnion fibroblast samples with or without cortisol treatment from three different TNL patients. Blue = low, red = high gene expression level

To identify the putative core transcription factor that is upregulated in parturition in a milieu rich in cortisol, transcriptome analysis was performed on the amnion tissue obtained from pregnant women undergoing spontaneous labor at term (term labor [TL]) or elective c section without labor at term (term non‐labor [TNL]) and on amnion fibroblasts prepared from the amnion obtained from TNL in the presence or absence of cortisol treatment. Transcriptome analysis revealed that there were 86 and 138 transcription factors that were significantly changed in the amnion tissue obtained from TL versus TNL and in amnion fibroblasts with or without cortisol treatment (1 μM for 24 h) (Figure 1). Further analysis of the transcriptome data revealed that all of the CEBP member transcripts were detectable except for *CEBPE* in either amnion fibroblasts or amnion tissue, and *CEBPD* was not only the most abundant C/EBP member in the amnion tissue and fibroblasts, but also the only member that was upregulated by both labor and cortisol treatment (Figure S1). Although *CEBPB* was increased in the amnion tissue by labor, it was not changed in amnion fibroblasts by cortisol treatment (Figure S1). The remaining members showed no changes either with labor or upon cortisol treatment (Figure S1).

### 2.2 Concurrent increases in C/EBPδ, 11β‐HSD1, and COX‐2 protein in the amnion tissue at parturition

**FIGURE 2:**
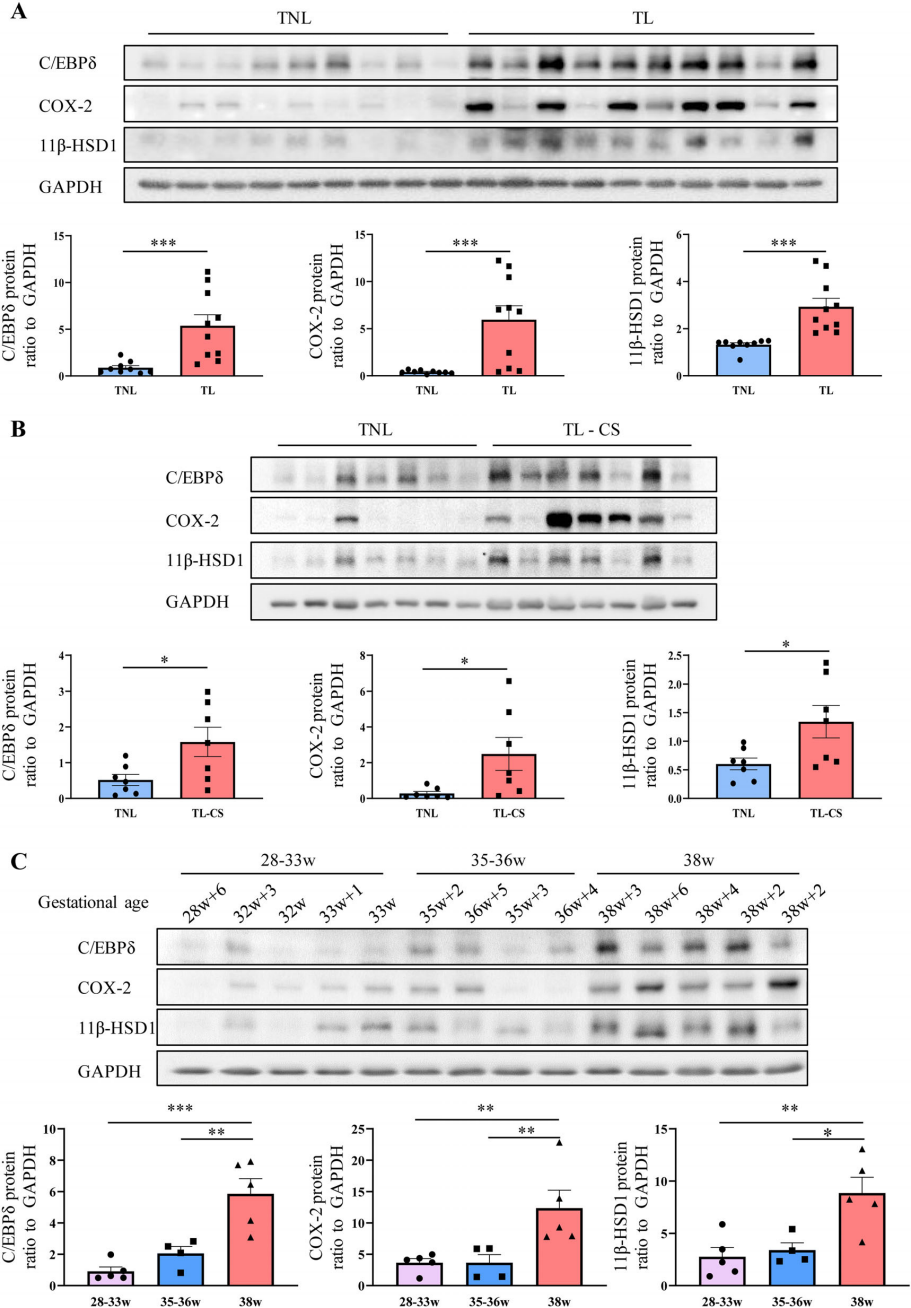
Changes of C/EBPδ, COX‐2 and 11β‐HSD1 protein abundance in human amnion in the 3rd trimester and at parturition. (A) C/EBPδ, COX‐2, and 11β‐HSD1 protein abundance in human amnion collected from term pregnancies without labor (TNL) (*n* = 9) and with labor (TL) (*n* = 10). (B) C/EBPδ, COX‐2, and 11β‐HSD1 protein abundance in human amnion collected from TNL (*n* = 7) and emergency c section after early onset of labor (TL‐CS) (*n* = 7). (C) C/EBPδ, COX‐2, and 11β‐HSD1 protein abundance in human amnion collected from c section without labor at 28w+6d to 38w+6d gestation. Western blots are shown above the mean data for protein abundance normalized to GAPDH. TNL samples in (A), (B), and (C) are from different subjects. Data are shown as mean ± SEM. *p < 0.05, **p < 0.01 (A and B: Mann‐Whitney test; C: one‐way ANOVA followed by the Newman‐Keuls multiple comparison test)

To confirm the above transcriptome findings, Western blotting analyses were conducted on the protein extracted from the amnion tissue collected from TL and TNL or emergency c section with early onset of labor (TL‐CS) at term. Women in the TL‐CS group received emergency c section after early onset of labor because of cephalopelvic disproportion and fetal distress. The demographic and clinical characteristics of recruited women are given in Tables S1A and S1B. The abundance of C/EBPδ protein significantly increased in the amnion tissue of both TL and TL‐CS groups as compared with that of the TNL group (Figures 2A and 2B), which was consistent with the mRNA data obtained from the transcriptome study. In addition to C/EBPδ protein, the abundance of 11β‐HSD1 and COX‐2 protein also significantly increased in the amnion tissue of both TL and TL‐CS groups versus TNL group (Figures 2A and 2B).

### 2.3 Increased C/EBPδ, 11β‐HSD1, and COX‐2 protein in amnion tissue before labor onset at term

To further investigate whether the abundance of C/EBPδ, 11β‐HSD1, and COX‐2 was already increased in the amnion before the onset of labor, the amnion tissue was collected from women undergoing c section without labor at gestational ages ranging from 28 weeks to term (38 weeks). C section before term was performed for the reasons of maternal cardiovascular disease (modified World Health Organization class III‐IV risks), antepartum hemorrhage (placenta previa or placenta accrete), scarred uterus (high risk of uterine rupture), and enormous sacrococcygeal benign tumor. The demographic and clinical characteristics of recruited women for this purpose are given in Table S1C. Western blotting showed that C/EBPδ, 11β‐HSD1, and COX‐2 protein all significantly increased in the amnion tissue at term (38 weeks) without labor compared to that before term at 28–36 weeks (Figure 2C), indicating that the abundance of C/EBPδ, 11β‐HSD1, and COX‐2 in the amnion increased at term before the onset of labor and further increased at parturition.

### 2.4 Distribution of C/EBPδ in the amnion

**FIGURE 3:**
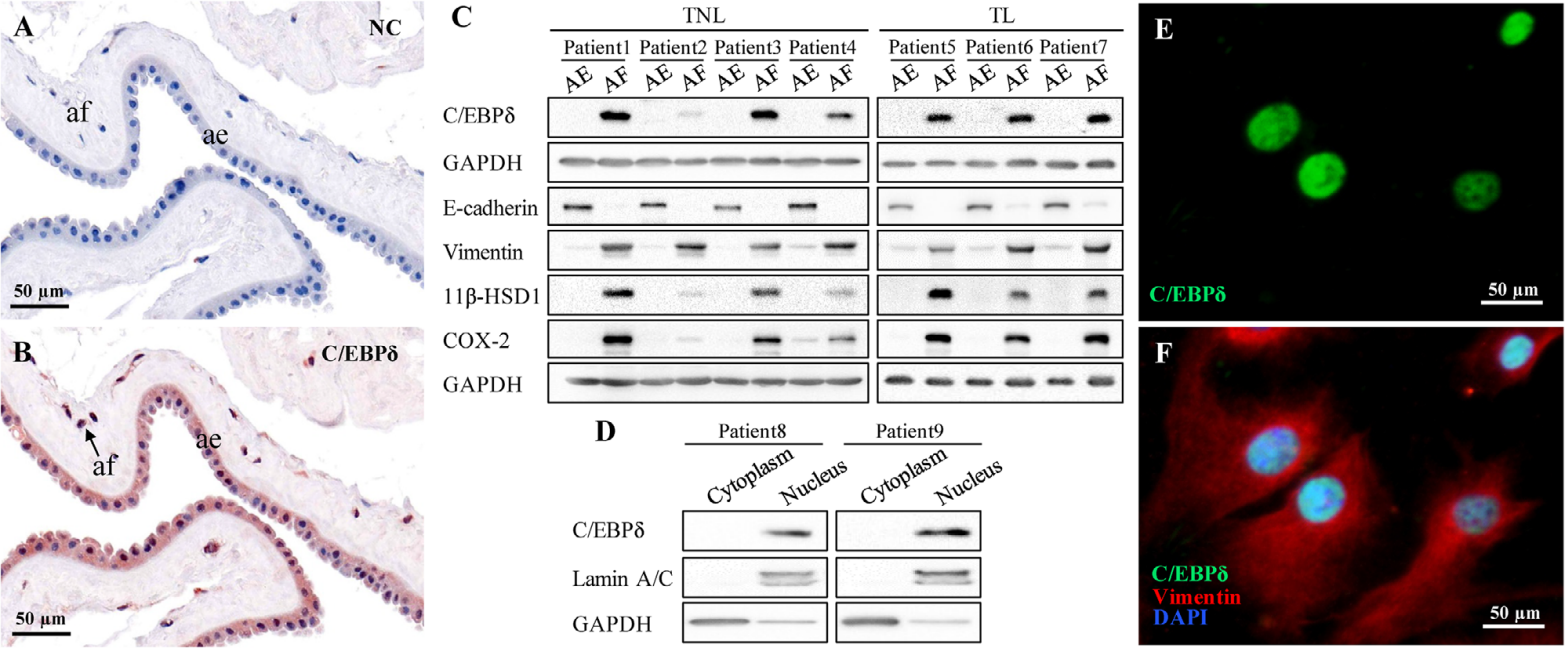
Distribution of C/EBPδ in human amnion tissue and fibroblasts. (A and B) Immunohistochemical staining of C/EBPδ in the human amnion obtained from elective c‐section at term without labor (TNL). Scale bars, 50 μm. Arrows indicate amnion fibroblasts. (C) Western blots showing C/EBPδ, COX‐2, and 11β‐HSD1 abundance in cultured primary amnion epithelial cells and fibroblasts prepared from four individual TNL patients and three individual term pregnancies with labor (TL) patients. E‐cadherin and vimentin served as markers for epithelial cells and fibroblasts, respectively. (D) Western blots showing C/EBPδ in the nuclear fractions in human amnion fibroblasts from two individual patients. GAPDH and lamin A/C served as markers for cytoplasm and nucleus respectively. (E and F) Immunofluorescent staining showing nuclear localization of C/EBPδ (green) in human amnion fibroblasts (marked by cytoplasmic vimentin staining [red] and nuclear DAPI staining [blue]). Scale bars, 50 μm. Abbreviations: ae, amnion epithelial cells; af, amnion fibroblasts; NC, negative control

To determine the specific amnion cell type for further analysis, we examined the distribution and abundance of C/EBPδ among them. Immunohistochemical staining of the amnion tissue collected from TNL revealed positive staining for C/EBPδ in both epithelial and fibroblast cells (Figures 3A and 3B). However, Western blotting analysis of the protein extracted from cultured cells showed that C/EBPδ, COX‐2, and 11β‐HSD1 were all more abundant in fibroblasts than in epithelial cells in both TNL and TL patients (Figure 3C). Further analysis of nuclear and cytoplasmic protein fractions obtained from cultured amnion fibroblasts showed that C/EBPδ localized in the nuclear fraction of the cells in tissues of both patients studied (Figure 3D). The nuclear localization of C/EBPδ in amnion fibroblasts was further confirmed by immunofluorescence staining (Figures 3E and 3F), consolidating its role in transcriptional regulation.

### 2.5 Induction of C/EBPδ expression by cortisol, PGE2, and IL‐1β in amnion fibroblasts

**FIGURE 4:**
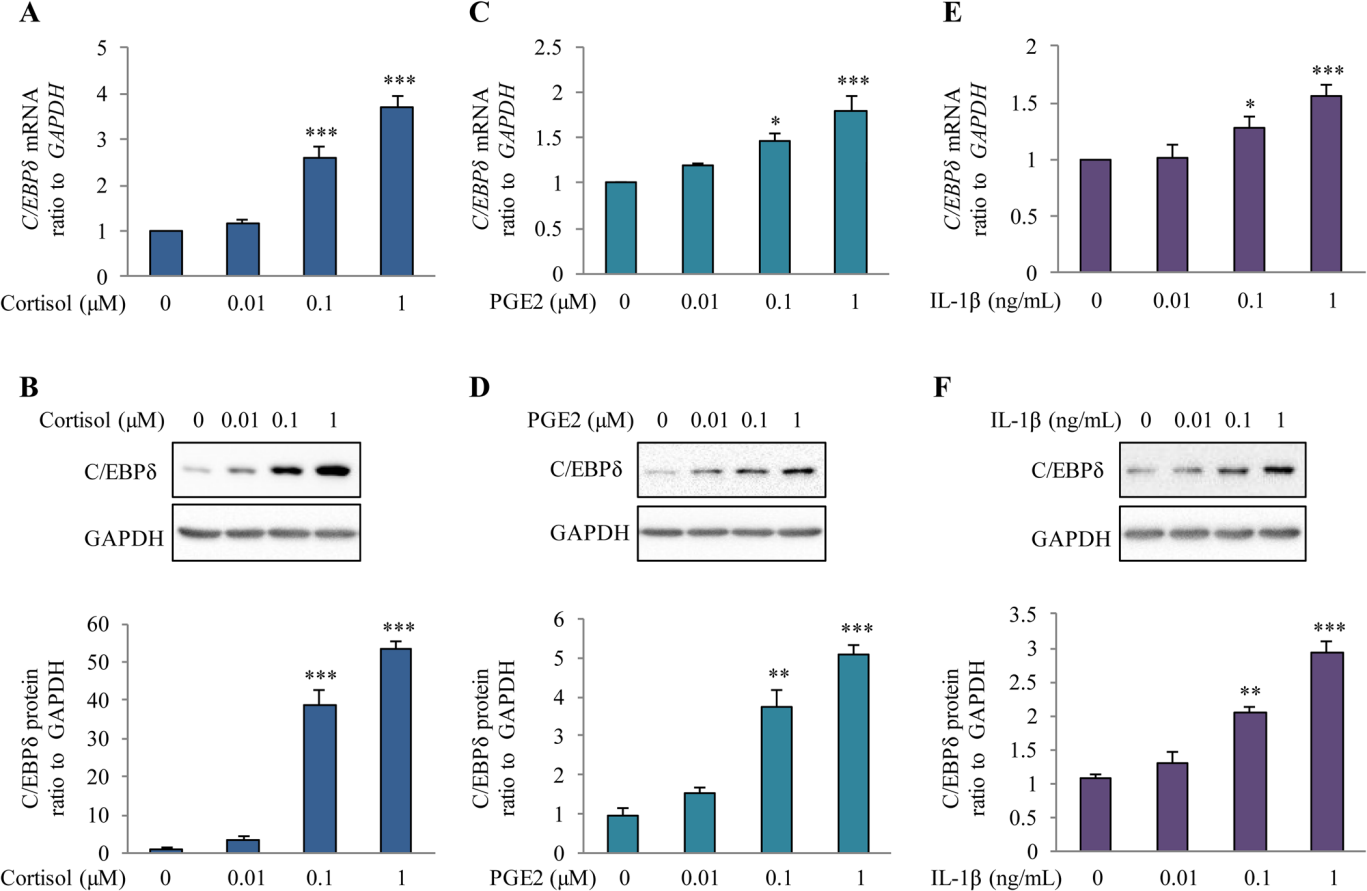
Effects of cortisol, PGE2, and IL‐1β on C/EBPδ abundance in human amnion fibroblasts. Concentration‐dependent effects of cortisol (0.01, 0.1, and 1 μM; 24 h), PGE2 (0.01, 0.1, and 1 μM; 24 h) and IL‐1β (0.01, 0.1, and 1 ng/ml; 24 h) on *CEBPD* mRNA (A, C, and E) and C/EBPδ protein abundance (B, D, and F). Data are means ± SEM from three to four experiments with representative Western blots shown above the mean data for protein abundance normalized to GAPDH. *p < 0.05, **p < 0.01, ***p < 0.001 versus untreated controls (one‐way ANOVA followed by the Newman‐Keuls multiple comparison test)

To further justify the choice of amnion cells for study, we prepared epithelial cells and fibroblasts from the amnion obtained from TNL to determine which cell type was responsive to cortisol treatment in terms of *CEBPD* expression. We found that cortisol (0.01, 0.1, and 1 μM; 24 h) increased *CEBPD* mRNA only in fibroblasts (Figure 4A) but not in epithelial cells (Figure S2), and this was confirmed at the protein level (Figure 4B). These data prompted us to use amnion fibroblasts to investigate whether PGE2 and interleukin‐1β (IL‐1β), both involved in parturition, were capable of inducing *CEBPD* expression as well. Like cortisol, PGE2 (0.01, 0.1 and 1 μM; 24 h), and IL‐1β (0.01, 0.1 and 1 ng/ml; 24 h) also increased the abundance of *CEBPD* mRNA and C/EBPδ protein in a concentration‐dependent manner (Figure 4C‐F) along with increased *PTGS2* and *HSD11B1* expression (Figure S3), suggesting that these factors may be, at least in part, responsible for the increases in C/EBPδ, COX‐2, and 11β‐HSD1 abundance in the amnion at term and at parturition. However, none of the other C/EBP members were changed by all three of cortisol, PGE2, and IL‐1β treatment of amnion fibroblasts (Figure S4). The expression of *CEBPB* and *CEBPG* mRNA was increased only by IL‐1β but not by cortisol or PGE2 (Figure S4). Moreover, instead of induction, the abundance of *CEBPG* or *DDIT3* mRNA was reduced by cortisol or PGE2 treatment of amnion fibroblasts (Figure S4), although only nonsignificant trends toward reduction were revealed upon cortisol treatment in the transcriptome study. These data suggest that C/EBPδ is more likely the universal transcription factor that may mediate simultaneous induction of 11β‐HSD1 and COX‐2 expression by the major known factors involved in parturition.

### 2.6 *PTGS2 and HSD11B1* were among the downstream target genes of C/EBPδ in amnion fibroblasts

**FIGURE 5:**
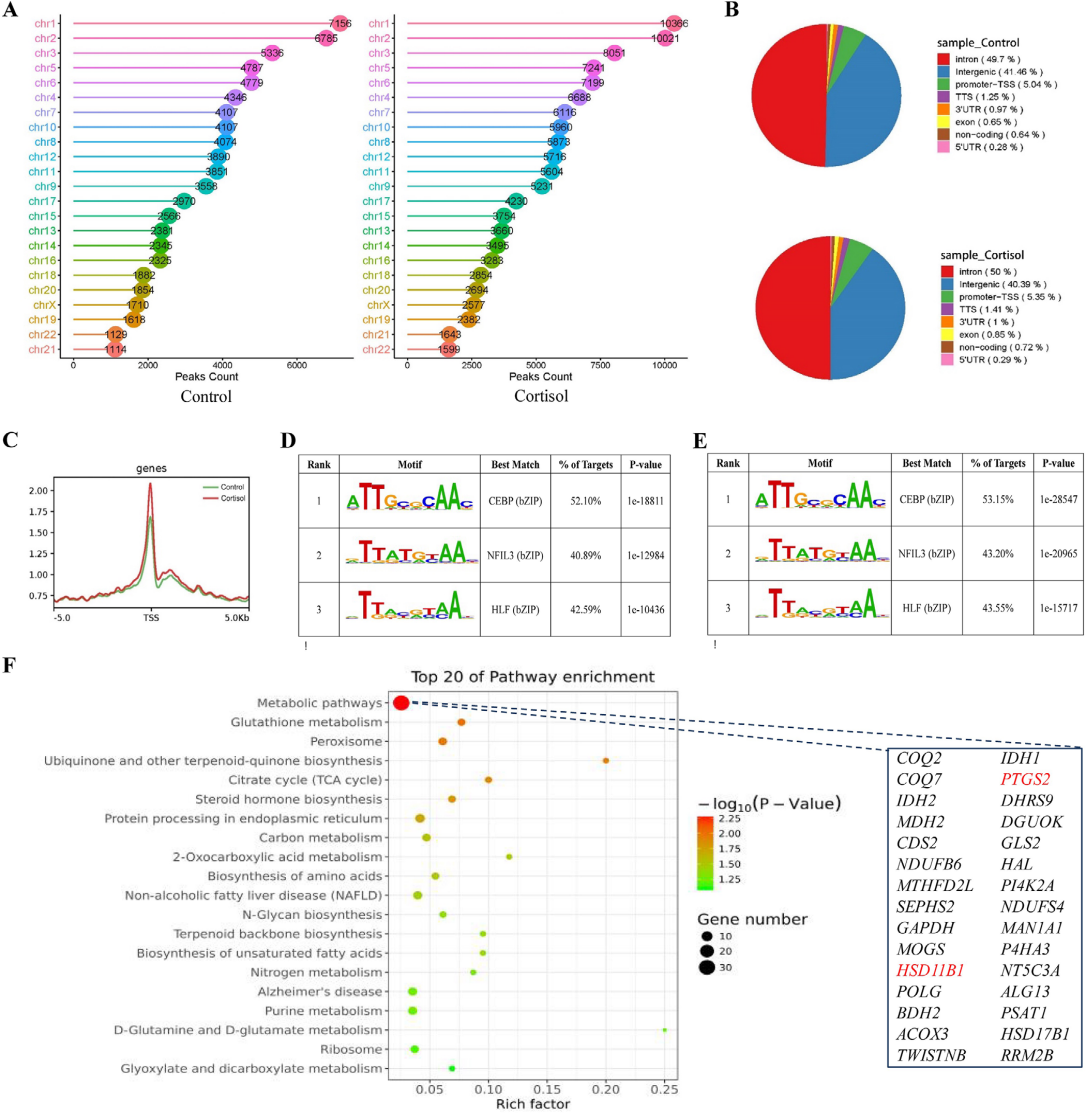
Analysis of putative downstream target genes of C/EBPδ with ChIP‐seq in human amnion fibroblasts with or without cortisol treatment. (A) Genomic distribution of the C/EBPδ ChIP‐seq peaks over the human genome in control (left) and cortisol‐treated (right) groups. (B) Pie diagram showing the percentage of the C/EBPδ‐binding sites located in the 5′ UTR, promoter‐TSS, TTS, exon, intron, 3′ UTR, intergenic region, and non‐coding region. The left and right panels represent control and cortisol‐treated groups, respectively. (C) Distribution of the C/EBPδ‐binding sites within a range of 5 kb up‐ and down‐streams of TSS point. TSS is defined as position zero in the graph. Y‐axis represents normalized tag density. Green and red lines represent control and cortisol‐treated groups, respectively. (D and E) Top three de novo motifs returned by Homer2 for all C/EBPδ binding sites in amnion fibroblasts without (D) or with (E) cortisol treatment. Percentage of binding sites and *p*‐value were calculated using the optimal motif score threshold as determined by Homer2. (F) KEGG pathway analysis of genes with increased C/EBPδ enrichment at the promoter after cortisol treatment (1 μM; 12 h). A bar plot was generated for the top 20 enriched KEGG terms with the most significant *p* values (Fisher's exact test). The gene list on the right panel is all genes in the metabolic pathway including *PTGS2* and *HSD11B1*, which are in red. Abbreviations: TTS, transcription termination site; TSS, transcription start site

**FIGURE 6:**
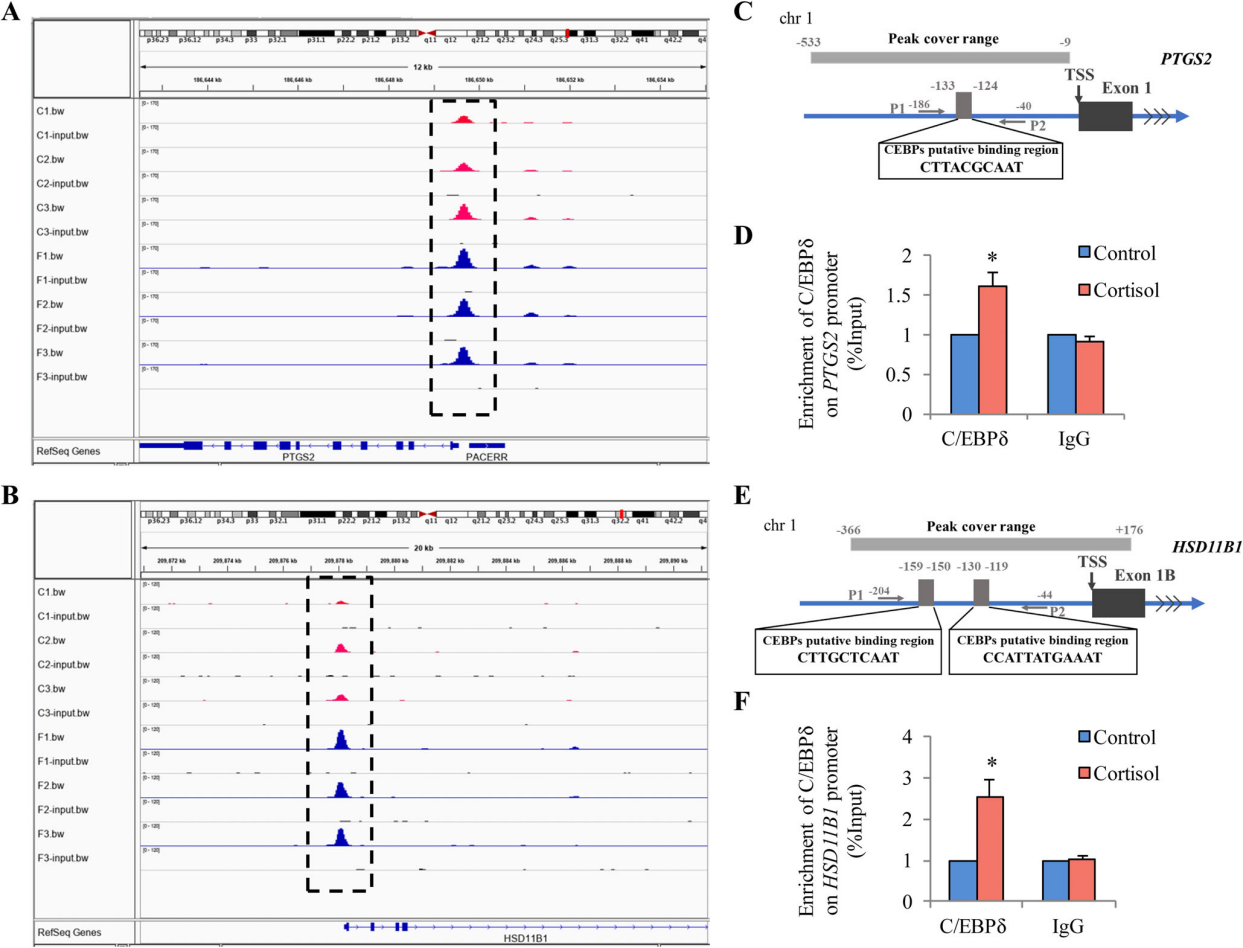
C/EBPδ enrichments at *PTGS2* and *HSD11B1* gene loci in human amnion fibroblasts with or without cortisol treatment. (A and B) Integrated genome browser visualization of C/EBPδ ChIP‐seq signal at *PTGS2* (A) and *HSD11B1* (B) loci in amnion fibroblasts without (red) and with (blue) cortisol (1 μM; 12 h) stimulation. Input as negative control sequencing. C1, C2, C3 (control group) and F1, F2, F3 (cortisol‐treated group) are paired samples from three different patients. (C and E) Diagrams showing putative binding sites for C/EBPδ in *PTGS2* (C) and *HSD11B1* (E) promoters. Arrows indicate primer aligning positions in ChIP assay. (D and F) ChIP assay for the enrichment of C/EBPδ at *PTGS2* (D) and *HSD11B1* (F) promoters in human amnion fibroblasts in response to cortisol treatment (1 μM; 12 h). IgG served as the negative control. Data are mean ± SEM from four experiments. *p < 0.05 versus control (paired Student's *t* test). Abbreviation: TSS, transcription start site

Next, in order to identify the downstream target genes to C/EBPδ signaling in amnion fibroblasts, chromatin immunoprecipitation sequencing (ChIPseq) was performed with or without cortisol treatment of the cells and upon precipitation with the C/EBPδ antibody. Because binding of the transcription factor to the gene promoter is a sequential event before gene expression, incubation of the cells with cortisol for 12 h rather than 24 h was adopted in ChIP assay. A number of genes were revealed as potential downstream targets of C/EBPδ in cortisol‐treated amnion fibroblasts. Global analysis of the ChIP‐seq data identified 78,670 peaks in the control group and 116,237 peaks in cortisol‐treated group using the model‐based analysis of ChIP‐seq (MACS) algorithm with *p* < 0.01, and the greatest number of peaks distributed in chromosome 1 (Figure 5A). The complete list of the peaks is provided in Dataset 2. The peaks were mapped to reference genes of NCBI GRCh38, and the peak distribution relative to the genomic boundaries was analyzed using Homer2.^45^ The results indicated that 5.04% (control) and 5.35% (cortisol) of the C/EBPδ‐binding sites were enriched within 1 kb upstream to 1 kb downstream of the promoter‐transcription start sites (TSS) region (Figure 5B). Within a range of 5 kb up‐ and down‐stream of the TSS, normalized tag (reads) which assembled near TSS showed a strong signal of C/EBPδ binding (Figure 5C). *De novo* motif analysis of peaks confirmed the enrichment of C/EBP binding sequences as the top motifs (Figures 5D and 5E). Kyoto encyclopedia of genes and genomes (KEGG) analysis of genes with significant C/EBPδ enrichments (≥1.5‐fold change) after cortisol treatment (Dataset 3) revealed genes in metabolic pathways as most significantly enriched (Figure 5F). There were 30 genes including *PTGS2* and *HSD11B1* in this pathway (Figure 5F). The peak spanning regions in *PTGS2* and *HSD11B1* promoters ranged from −533 to −9 bp and from −366 bp to +176, respectively (Figures 6A and 6B). C/EBPδ enrichments at these regions of *PTGS2* and *HSD11B1* promoters were confirmed by ChIP assay in amnion fibroblasts treated with cortisol using primers spanning the regions of −186 to −40 bp for *PTGS2* and −204 to −44 bp for *HSD11B1*, respectively, which carry C/EBP binding sites (Figures 6C‐6F). Of interest, ChIP‐seq revealed that glyceraldehyde 3‐phosphate dehydrogenase (*GAPDH)* was also among the genes with increased enrichment of C/EBPδ after cortisol treatment (Figure 5F). However, measurements with qRT‐PCR and Western blotting showed no changes in its abundance at both mRNA and protein levels upon cortisol treatment of amnion fibroblasts (Figure S5), which validated its use as a reference house‐keeping gene in this study.

### 2.7 Attenuation of 11β‐HSD1 and COX‐2 induction by cortisol and PGE2 with knock‐down of *CEBPD by siRNA*

**FIGURE 7:**
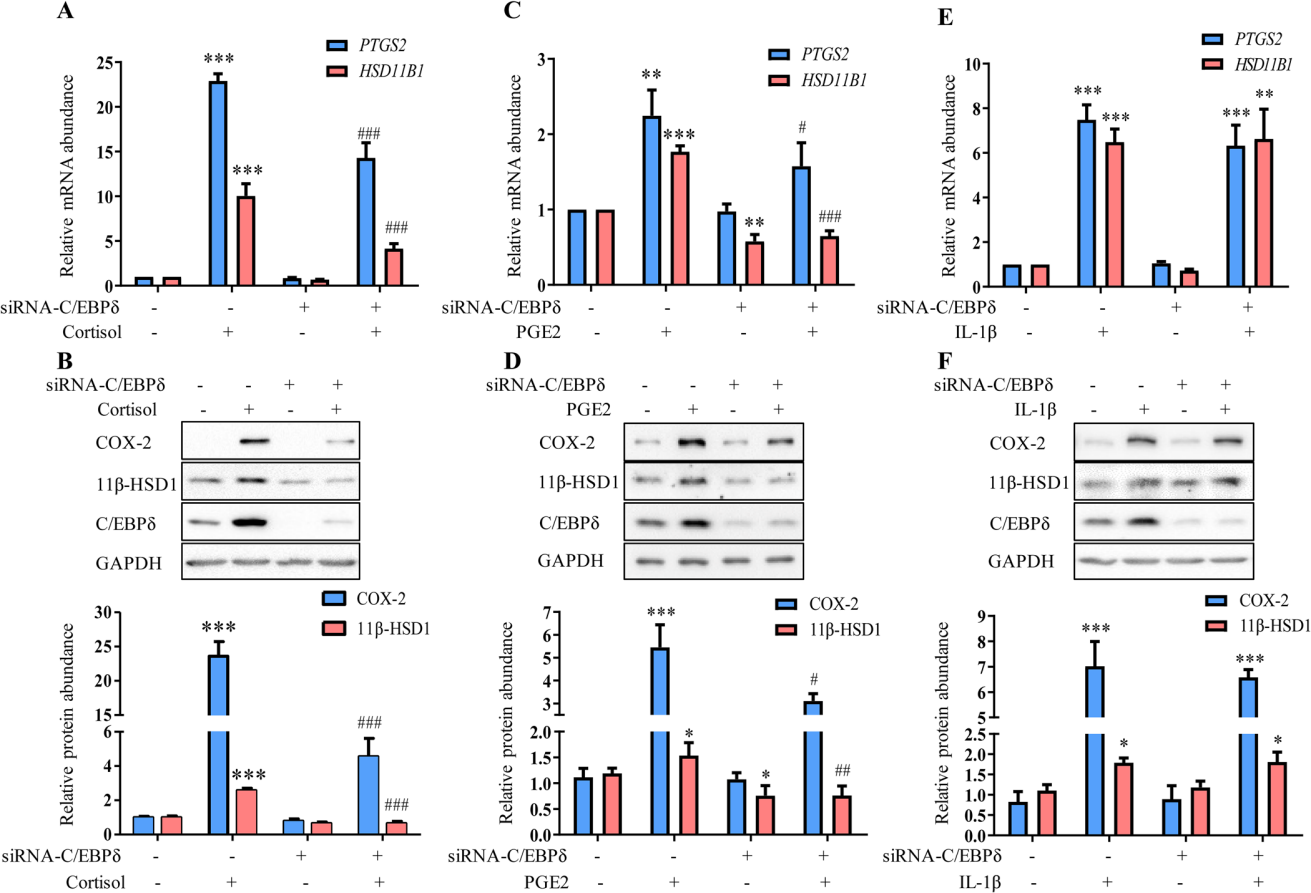
Role of C/EBPδ in the induction of COX‐2 and 11β‐HSD1 expression by cortisol, PGE2 and IL‐1β in human amnion fibroblasts. (A‐E) Quantification of *PTGS2* and *HSD11B1* mRNA as well as COX‐2 and 11β‐HSD1 protein in cortisol (1 μM; 24 h) (A and B), PGE2 (1 μM; 24 h) (C and D) and IL‐1β (1 ng/ml; 24 h) (E and F)‐treated human amnion fibroblasts transfected with scrambled (−) or C/EBPδ‐targeted (+) siRNA. Data are mean ± SEM from three to four experiments with representative Western blots shown above the mean data for protein abundance normalized to GAPDH. *p < 0.05, **p < 0.01, ***p < 0.001 against control with scrambled siRNA; #*p* < 0.05, ##*p* < 0.01, ###*p* < 0.001 compared to cells treated with cortisol, PGE2 or IL‐1β (one‐way ANOVA followed by the Newman‐Keuls multiple comparison test)

To further investigate the role of C/EBPδ in the regulation of 11β‐HSD1 and COX‐2 expression by cortisol, PGE2, and IL‐1β, knock‐down of *CEBPD* by siRNA was performed in cultured amnion cells. The data showed that the increased 11β‐HSD1 and COX‐2 abundance in amnion fibroblasts by cortisol (1 μM; 24 h) and PGE2 (1 μM; 24 h) were all significantly attenuated with knock‐down of *CEBPD* by siRNA (Figures 7A‐7D), which was confirmed by another siRNA against *CEBPD* (Figure S6). Of interest, the increased 11β‐HSD1 and COX‐2 abundance with addition of IL‐1β was not affected by knock‐down of *CEBPD* by siRNA (Figures 7E and 7F), suggesting that unlike the dominant role of C/EBPδ in the induction of 11β‐HSD1 and COX‐2 by cortisol and PGE2, an alternative transcriptional mechanism may operate in the induction of 11β‐HSD1 and COX‐2 by IL‐1β.

### 2.8 Role of C/EBPδ in parturition in the mouse

**FIGURE 8:**
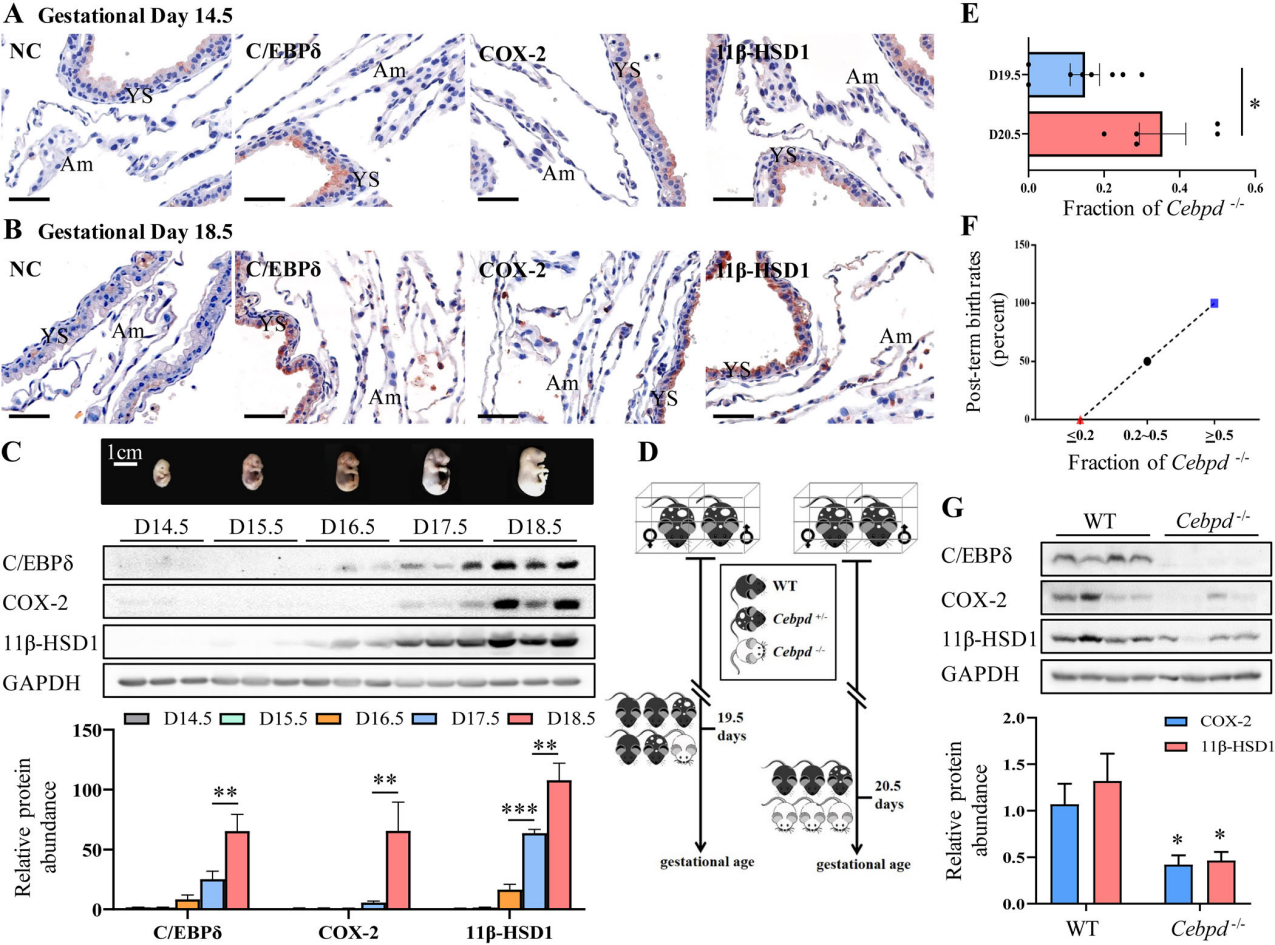
C/EBPδ in the fetal membranes of C57BL/6 mice and its role in parturition. (A and B) Immunohistochemical staining of C/EBPδ, COX‐2 and 11β‐HSD1 in mouse fetal membranes obtained on gestational day 14.5 (A) and 18.5 (B) without labor. Scale bars, 50 μm. (C) Western blots showing the abundance of C/EBPδ, COX‐2, and 11β‐HSD1 protein in the fetal membranes from day 14.5 to day 18.5. Images of mouse embryos corresponding to gestational ages are shown above. The mean data for protein abundance normalized to GAPDH are shown below. Data are shown as mean ± SEM. *p < 0.05, ***p < 0.001 (one‐way ANOVA followed by the Newman‐Keuls multiple comparison test). (D) Schematic diagram showing the breeding strategy. (E) The fraction of homozygous embryos (*Cebpd* ^−/−^) in term (19.5 days) and post‐term (20.5 days) dams. Data are shown as mean ± SEM. *p < 0.05 (unpaired Student's *t* test). (F) Correlation between fraction of *Cebpd* ^−/−^ embryos and post‐term birth rate. (G) Western blots showing C/EBPδ, COX‐2, and 11β‐HSD1 protein abundance in the fetal membranes collected from WT (*n* = 4) and *Cebpd* ^−/−^ (*n* = 4) embryos. Data are shown as mean ± SEM. *p < 0.05 versus WT (unpaired Student's *t* test). Abbreviations: Am, amnion; *Cebpd* ^+/−^, heterozygous mice; *Cebpd* ^−/−^, homozygous mice; NC, negative control; WT: wild‐type mice; YS, yolk sac.

The role of C/EBPδ in parturition was further investigated in both wild‐type and *Cebpd* knockout mice. The mouse fetal membranes are formed by the amnion and yolk sac membranes. Immunohistochemical staining of day 14.5 and 18.5 wild‐type mouse fetal membranes showed the presence of C/EBPδ, 11β‐HSD1, and COX‐2 in both amnion and yolk sac membranes, with abundance appearing to increase from day 14.5 to 18.5 (Figures 8A and 8B), which was confirmed by ontogenic analysis with Western blotting (Figure 8C), showing that the protein abundance of C/EBPδ, 11β‐HSD1, and COX‐2 increased from day 17.5 onwards (Figure 8C). To further examine the role of C/EBPδ in mouse parturition, *Cebpd* knockout mice were generated using a CRISPR/Cas9 system. More details of the breeding strategy are given in the section of materials and methods. Gestational length was not changed when wild‐type mice were crossed with heterozygous (*Cebpd* ^+/−^) mice, which produced only wild‐type or heterozygous (*Cebpd* ^+/−^) embryos, suggesting that heterozygous (*Cebpd* ^+/−^) embryos do not change the gestational length. However, gestational length was prolonged to 20.5 days in some of the *Cebpd* ^+/−^ dams that crossed with *Cebpd* ^+/−^ males (Figure 8D). Genotype analysis revealed that those dams with prolonged gestational length had a significantly higher fraction of *Cebpd* ^−/−^ embryos than the dams that delivered on time at normal term (19.5 days) (Figure 8E). Further analysis revealed a positive correlation between the fraction of *Cebpd* ^−/−^ embryos and the rate of post‐term birth (Figure 8F). Western blotting analysis showed corresponding significant decreases in COX‐2 and 11β‐HSD1 protein abundance in the fetal membranes of *Cebpd* ^−/−^ embryos (Figure 8G). However, there was no significant difference in the litter size between post‐term and normal‐term groups (Figure S7). The information on pregnancy, delivery time, and offspring genotypes in crosses between *Cebpd* ^+/−^ mice is provided in Table S2.

## 3 DISCUSSION

Hormones synthesized in the fetal membranes rely heavily on diffusion to target cells as the membranes are not vascularized.^2^, ^46^ Therefore, local actions of these membrane‐derived hormones on intrauterine tissues account for most of their effects in parturition. The actions need to be coordinated to allow parturition to proceed in a feed‐forward manner. The mutually enhancing pattern of 11β‐HSD1 and COX‐2 expression in the amnion illustrates such concerted coordination,^13^ which is known to culminate in the feedforward accumulation of cortisol and PGE2/PGF2α in the fetal membranes toward the end of gestation.^7^, ^13^ The mutual induction of 11β‐HSD1 and COX‐2 by cortisol and PGE2 was recapitulated in vitro in amnion fibroblasts in the present study. Given the important actions of PGE2/PGF2α and cortisol in parturition, identifying the common transcription factor driving both 11β‐HSD1 and COX‐2 expression in the fetal membranes may provide a potential therapeutic target for the regulation of both cortisol and PGE2/PGF2α productions in pre‐term birth. The present study, for the first time, describes C/EBPδ as a common transcription factor employed by both glucocorticoids and PGs in the induction of both 11β‐HSD1 and COX‐2 in amnion fibroblasts. The crucial role of C/EBPδ in parturition is illustrated by its significant increase in the amnion tissue in human labor as well as by the delay in parturition seen in the mouse when the fraction of homozygous (*Cebpd* ^−/−^) embryos is high. Similar to human fetal membranes, 11β‐HSD1, COX‐2, and C/EBPδ were all expressed in the mouse fetal membranes, and increased in late gestation, suggesting that increased expression of these genes is a requisite event for the onset of labor. As all the maternal genotypes are heterozygotes (*Cebpd* ^+/−^) in this study, the delay of parturition was apparently a consequence of offspring but not maternal genotype. Since systemic *Cebpd* knockouts were used in this study, it remains to be determined, however, whether C/EBPδ in non‐fetal membrane tissue of the fetus may also contribute to the process of parturition.

C/EBPs play pivotal roles in a number of cellular responses including cell proliferation, metabolism differentiation, and inflammatory responses.^48^ In inflammatory responses, the expression of C/EBPs, particularly C/EBPβ and δ, is induced by a number of inflammatory factors including lipopolysaccharides and proinflammatory cytokines.^49^, ^50^, ^51^, ^52^ Binding sites for C/EBPs have been identified in the regulatory regions of a number of inflammatory genes including *PTGS2*.^41^, ^42^, ^53^, ^54^, ^55^, ^56^, ^57^, ^58^ However, in contrast to the consistent upregulation of 11β‐HSD1 by C/EBP members,^31^, ^32^, ^33^, ^34^ the regulation of COX‐2 by C/EBPs appears to be cell‐context dependent, which can be either suppressive or stimulatory.^41^, ^42^, ^43^, ^44^ In this study, we also found that C/EBPδ could be induced not only by glucocorticoids but also by proinflammatory factors such as PGE2 and IL‐1β, and C/EBPδ mediated the induction of not only 11β‐HSD1 but also COX‐2 expression in amnion fibroblasts. It is now widely accepted that labor is a process of inflammation of the gestational tissues no matter in the presence or absence of infection.^59^, ^60^ This study provides evidence that C/EBPδ is likely to be involved in the inflammatory process of the fetal membranes at parturition by regulating key reactions leading to PG accumulation. It has been reported that the response of C/EBPs usually occurs later than the transient responses of NFκB and STAT3 to inflammatory stimuli.^57^ Thus, it is proposed that C/EBPs may be responsible for the delayed and long‐lasting responses, and NFκB and STAT3 may be responsible for the early responses to inflammatory stimuli.^57^ Our previous studies have demonstrated that glucocorticoids and PGs are capable of inducing STAT3 phosphorylation in amnion fibroblasts.^19^, ^27^, ^47^ It has been shown that the transcription of the *CEBPD* gene can be induced upon STAT3 phosphorylation with its subsequent binding to the promoter region of *CEBPD* in mammary epithelial cells and hepatocytes.^61^, ^62^, ^63^ Thus, it is possible that STAT3 mediates the early while C/EBPδ mediates the late inflammatory responses respectively in the fetal membranes. Further temporal and spatial studies on total or phosphorylated STAT3, CREB, and C/EBPδ abundance in amnion fibroblasts should help to resolve this issue.

There are at least six members in the C/EBP family.^39^, ^64^ Although all C/EBP members share substantial sequence homology in their C‐termini, the N‐termini of C/EBP proteins are quite divergent.^48^, ^64^ The C‐terminal is responsible for DNA binding and dimerization, while the N‐terminal is responsible for transactivation. Dimerization of C/EBP proteins (either homodimer or heterodimer) is a prerequisite for DNA binding.^65^, ^66^ However, the transcriptional activity of the heterodimer is dependent on the N‐terminal structure of its partner.^48^, ^64^ If the partner C/EBP protein lacks the activation domain in the N‐termini, a dominant negative heterodimer is then formed as an inhibitor of C/EBP transcriptional activity. C/EBPγ, the 30 kDa isoform of C/EBPα, and the 20 kDa isoform of C/EBPβ, also known as the liver‐enriched inhibitory protein are such C/EBP proteins without the activation domain or with low activation activity.^67^, ^68^, ^69^ Therefore, the expression profile of each C/EBP family member in a particular cell may determine the functional properties of C/EBP dimers. This study revealed that the most abundant C/EBP member in amnion tissue and fibroblasts is C/EBPδ, followed by C/EBPβ, C/EBPζ, C/EBPγ, and C/EBPα with barely detectable C/EBPε. Although C/EBPδ was shown to be the dominant member mediating the induction of COX‐2 and 11β‐HSD1 by cortisol and PGE2 in amnion fibroblasts, it does not necessarily mean that other members are not involved under particular conditions. Indeed, we found that in addition to increased C/EBPδ abundance, the abundance of *CEBPG* or *DDIT3* mRNA was reduced with cortisol or PGE2 treatment of amnion fibroblasts. Given that C/EBPγ may form dominant negative heterodimers with other members, it is likely that the simultaneous inhibition of *CEBPG* or *DDIT3* expression is necessary for the transcriptional activity of C/EBPδ in this study. Of interest, IL‐1β was the only factor tested which did not affect the expression of *CEBPG* or *DDIT3* but induced both C/EBPβ and C/EBPδ in amnion fibroblasts in this study. The differential expression profile of C/EBP members upon IL‐1β treatment may account for the failure of blockade of IL‐1β‐induced COX‐2 and 11β‐HSD1 expression when knocking‐down C/EBPδ. Moreover, the induction of *CEBPB* by IL‐1β but not by cortisol and PGE2 in amnion fibroblasts may also explain why our transcriptome study revealed increased abundance of *CEBPB* in the amnion tissue following spontaneous labor but not in amnion fibroblasts with cortisol treatment.

In conclusion, we have demonstrated in this study that C/EBPδ is a crucial transcription factor in amnion fibroblasts driving at least two inter‐related key endocrine events in parturition, that is, cortisol regeneration and PG synthesis. Moreover, the expression of C/EBPδ can be induced by well‐described major parturition‐promoting factors including glucocorticoids and PGs. Therefore, C/EBPδ may hold the key to membrane activation in parturition, and targeting the C/EBPδ pathway may be speculated to have potential value in the treatment of pre‐term birth. In addition to *HSD11B1* and *PTGS2*, a wealth of other genes has also been revealed as potential target genes for C/EBPδ in amnion fibroblasts by ChIP‐seq, suggesting that investigation of whether these potential target genes are also involved in parturition would be of interest.

## 4 MATERIALS AND METHODS

### 4.1 Collection of the human amnion

Fetal membranes were collected from pregnant women following a protocol approved by the Ethics Committee of Ren Ji Hospital, Shanghai Jiao Tong University School of Medicine, with written informed consent (Protocol No. [2013] N025). Pregnancies with complications such as preeclampsia, gestational diabetes, fetal growth restriction, and chorioamnionitis were not included in this study. The amnion within the cervical zone (site of membrane rupture) was immediately separated from the chorion/decidua for subsequent studies on the amnion tissue. The entire amnion from the reflected membranes was used for preparation of amnion cells. Details on labor status and gestational ages are described in corresponding sections.

### 4.2 Identification of the transcription factor regulated by both labor and cortisol with RNA sequencing

The fetal membranes were collected from spontaneous labor at term (TL) (*n* = 3) and elective c section without labor at term (TNL) (*n* = 3), and the amnion within the cervical zone was immediately separated from the chorion/decidua for RNA extraction using Trizol reagent (Life Technologies Inc., Grand Island, NY, USA). RNA was also extracted from cultured primary amnion fibroblasts prepared from the TNL amnion treated with or without (*n* = 3) each cortisol (1 μM; 24 h). The purity and integrity of extracted RNA were determined using a NanoDrop ND‐2000 and an Agilent 2200 TapeStation. RNA‐sequence libraries were constructed using a TruSeq RNA sample preparation kit (Illumina Inc.) according to manufacturer's protocol. The sequencing libraries were constructed on an IlluminaHiSeqTM 2500 system, and the RNA‐sequence data were processed using a computational pipeline. The clean reads were aligned to human genome (GRCh38, NCBI) using Hisat2 after removing the adaptor sequences and low‐quality reads.^70^ High‐throughput sequencing (HTseq)^71^ was used to get gene counts, and reads per kilo base per million mapped reads (RPKM) were used to determine gene expression levels. Differential expression was analyzed using the DESeq2 algorithm.^72^ For analysis of statistical significance, *p*‐value and false discovery rate (FDR) analysis were subjected to fold change ≥ 1.5 or ≤ 0.667, and *p*‐value and FDR < 0.05. In order to screen out the transcriptional factors, differentially expressed genes were intersected with a transcriptional factor database (Animal TFDB 3.0).^73^

### 4.3 Confirmation of RNA sequencing data and observation of gestational changes with Western blotting

To confirm the RNA sequencing data, the amnion tissues from TNL (*n* = 9) and TL (*n* = 10) were sampled within 5 cm of the cervical zone. To study whether the observed changes occurred with early labor onset at term, the amnion tissues were also collected from pregnant women undergoing emergency c section after early labor onset at term (TL‐CS, *n* = 7) for reasons of cephalopelvic disproportion and fetal distress. A different set of amnion tissues from TNL (*n* = 7) were collected as controls in the latter study. To examine gestational changes of studied proteins, amnion tissues were collected from pregnant women undergoing c section without labor at gestational ages 28–33 weeks (*n* = 5), 35–36 weeks (*n* = 4), and at term (38 weeks [*n* = 5]). C section before term was performed due to maternal cardiovascular disease (modified World Health Organization class III‐IV risks), antepartum hemorrhage (placenta previa or placenta accrete), scarred uterus (high risk of uterine rupture), or an enormous sacrococcygeal benign tumor.

The amnion tissue was homogenized in ice‐cold radio‐immunoprecipitation assay lysis buffer (Active Motif, Carlsbad, CA, USA) containing protease inhibitors (Roche, Indianapolis, IN, USA). After centrifugation, supernatants containing extracted protein were collected, and protein concentration was determined using the Bradford method. Sample protein (30 μg) was electrophoresed in a sodium dodecyl sulfate‐polyacrylamide gel. After transferring to a nitrocellulose membrane blot, the blot was blocked with 5% non‐fat milk and incubated with antibodies against C/EBPδ (1:1000; GeneTex; San Antonio, TX, USA), 11β‐HSD1 (1:1000; Abcam, Cambridge, MA, USA), and COX‐2 (1:2000; Cell Signaling Technology, Danvers, MA, USA), and then incubated with secondary antibodies conjugated with horseradish peroxidase. Chemiluminescence detection system (Millipore, Billerica, MA, USA) was used to develop the bands with peroxidase activity, and the bands were visualized using a G‐Box chemiluminescence image capture system (Syngene, Cambridge, UK). Band intensities were measured using gel‐pro analyzer software. Internal loading controls were performed by probing the same blot with a housekeeping gene GAPDH antibody (1:10000, Proteintech, Wuhan, China). The ratio of band intensities of C/EBPδ, COX‐2, and 11β‐HSD1 to that of GAPDH was used to indicate protein abundance. All the antibody information is illustrated in Table S3.

### 4.4 Observation of C/EBPδ distribution in amnion tissue and cells with immunohistochemical/immunofluorescent staining and Western blotting

To observe the distribution of C/EBPδ protein in amnion cells, paraffin‐embedded amnion tissue from TNL was sectioned for immunohistochemical staining. After quenching the endogenous peroxidase activity and blocking with normal serum, a primary antibody against C/EBPδ (1:50, GeneTex) was applied to the section with non‐immune serum served as negative control. After washing, a secondary antibody conjugated with biotinylated horseradish peroxidase was then applied to the section for further incubation. The peroxidase activity was developed using substrate 3‐amino‐9‐ethyl carbazole (Vector Laboratories, Burlingame, CA, USA) as a red color. The slide was counterstained with hematoxylin and examined under a microscope. The abundance of C/EBPδ in amnion fibroblasts and epithelial cells prepared from TNL patients were further examined along with 11β‐HSD1 and COX‐2 with Western blotting. Vimentin (1:10000; Santa Cruz, Dallas, TX, USA) and E‐cadherin (1:1000; Cell Signaling Technology) antibodies were used for probing vimentin and E‐cadherin as markers for mesenchymal cells and epithelial cells, respectively. For observation of subcellular distribution of C/EBPδ protein, cultured amnion fibroblasts prepared from the TNL amnion were fixed in 4% paraformaldehyde and then permeabilized for immunofluorescence staining. After blocking with normal serum, the cells was incubated with antibodies against C/EBPδ (GeneTex) at 1:50 dilution and vimentin (Santa Cruz) at 1:200 dilution, followed by incubation with secondary antibodies conjugated with Alexa Fluor 488 (green color) or Alexa Fluor 594 (red color) (Proteintech) respectively. Nuclei were visualized with DAPI (1 μg/ml, blue color) staining. A fluorescence microscope (Zeiss) was used to observe immunofluorescence staining. The nuclear and cytoplasmic protein fractions were isolated from amnion fibroblasts using a Nuclear Extract Kit (Active Motif) for further examination of C/EBPδ subcellular distribution with Western blotting with GAPDH and lamin A/C as cytoplasmic and nuclear markers, respectively.

### 4.5 Isolation and culture of human amnion fibroblasts and epithelial cells

Human amnion fibroblasts and epithelial cells were prepared from the TNL amnion for functional and regulatory studies of the target proteins as described previously.^74^ The amnion tissue was digested twice with 0.125% trypsin (Life Technologies Inc.) and then washed thoroughly with normal saline. The epithelial cells were collected from trypsin digestion and normal saline washes by centrifugation. The remaining amniotic tissue was further digested with 0.1% collagenase (Sigma, St. Louis, MO, USA) for isolation of fibroblast cells which were collected by centrifugation. Isolated fibroblasts and epithelial cells were cultured in Dulbecco's Modified Eagle Medium (DMEM) containing 10% fetal bovine serum (FBS) and antibiotics (all Life Technologies Inc.) for 3 days before any processing including immunofluorescence staining and drug treatments. This method of cell isolation produced high purity of epithelial cells (>99%) and fibroblasts (>95%), as we have previously described by staining the cells for cytokeratin, vimentin and CD45, markers for epithelial, mesenchymal, and hemopoietic cells, respectively.^16^, ^29^

### 4.6 Treatment of human amnion fibroblasts

Amnion epithelial cells and fibroblasts were treated in phenol red‐ and FBS‐free culture medium. For RNA sequencing, fibroblasts were treated with or without cortisol (1 μM; Sigma; 24 h). To determine whether cortisol affected the expression of *CEBPD* in amnion epithelial cells, the epithelial cells were treated with cortisol (0.01–1 μM; 24 h). To examine whether cortisol, PGE2, and IL‐1β affected the expression of *CEBPD, CEBPA, CEBPB, CEBPG, DDIT3, PTGS2*, and *HSD11B1* amnion fibroblasts, the cells were treated with cortisol (0.01–1 μM), PGE2 (0.01–1 μM; Cayman Chemicals, Ann Arbor, MI, USA), and IL‐1β (0.01–1 ng/ml; Sigma) for 24 h. To study the role of C/EBPδ in the regulation of COX‐2 and 11β‐HSD1 expression by cortisol, PGE2, and IL‐1β in amnion fibroblasts, the cells were treated with cortisol (1 μM) or PGE2 (1 μM) or IL‐1β (1 ng/ml) in the presence or absence of knockdown of C/EBPδ by siRNA for 24 h.

### 4.7 RNA and protein extraction from human amnion cells for qRT‐PCR and Western blotting

Extraction of RNA and protein from the above‐treated amnion cells was performed using total RNA isolation kit (Foregene, Chengdu, China) and RIPA lysis buffer (Active Motif) containing protease inhibitors (Roche). Complementary DNA (cDNA) was transcribed reversely from mRNA using a PrimeScript RT Master Mix Perfect Real Time Kit (Takara, Kyoto, Japan) for the measurement of the mRNA abundance of *CEBPA*, *CEBPB*, *CEBPD*, *CEBPG*, *DDIT3*, *PTGS2*, and *HSD11B1* with qRT‐PCRT using a power Sybr Premix Ex TaqTM kit (Takara) as previously described.^27^ *GAPDH* or *ACTB* was measured in parallel for normalization. The abundance of mRNA was quantified using the 2‐^△△Ct^ method. The primer sequences are illustrated in Table S4. The protein abundance of C/EBPδ, COX‐2, and 11β‐HSD1 was examined with Western blotting as described above. The ratio of band intensities of C/EBPδ, COX‐2, and 11β‐HSD1 over that of GAPDH was used as an indication of C/EBPδ, COX‐2, and 11β‐HSD1 protein abundance, respectively. The ratio of band intensities of GAPDH over that of β‐actin was calculated as a measure of GAPDH protein abundance.

### 4.8 Transfection of siRNA in amnion fibroblasts with electroporation

After isolation, amnion fibroblasts were transfected with 50 nM of siRNA against *CEBPD* (5′‐ACAGCCUGGACUUACCACCACUAAA‐3′) (GenePharma, Shanghai, China) (Figure 7) in Opti‐MEM (Life Technologies Inc.) using an electroporator (Nepa Gene, Chiba, Japan) at 175V for 5 ms following a previously described protocol.^27^ To confirm the specific effect of siRNA‐mediated knockdown, amnion fibroblasts were also transfected with a separate siRNA against *CEBPD* (5′‐CCCUUUGUAUUGUAGAUAATT‐3′) (Figure S6). For negative control, randomly scrambled siRNA (5′‐UUCUCCGAACGUGUCACGUTT‐3′) was used. The cells were then incubated in DMEM containing 10% FBS andantibiotics for 3 days before treatments. The knockdown efficiency was assessed with qRT‐PCR and Western blotting.

### 4.9 ChIP‐seq to identify the downstream target genes of C/EBPδ in amnion fibroblasts

To identify the downstream target genes of C/EBPδ, ChIP‐seq was performed on cultured amnion fibroblasts treated without (*n* = 3) or with (*n* = 3) cortisol (1 μM) for 12 h as described previously.^74^ Briefly, after cross‐linking with 1% formaldehyde, the cells were lysed with 1% sodium dodecyl sulfate buffer containing protease inhibitors. The chromatin DNA of the lysed cells was sheared to sizes around 500 bp with sonication, which was then precipitated with a C/EBPδ antibody (GeneTex). Western blotting showed that this antibody identified a single protein band around 36 kDa (Figure S8), suggesting that this antibody was quite specific. The immune complex precipitated by the C/EBPδ antibody was pulled down with protein A+G agarose magnetic beads (Millipore). After reverse cross‐linking, the sample was digested with proteinase K and RNase to remove protein and RNA and then subjected to DNA extraction for subsequent ChIP sequencing. An input sample without antibody precipitation was used as a negative control for noise elimination. The sequencing library generated using 5 ng of extracted DNA with a NEB kit for sequencing with the Illumina HiSeq X Ten. Raw sequence reads were initially processed by FastQC for quality control. After removal of adapter sequences and poor‐quality reads, filtered quality reads were mapped to reference genome using Bowtie2^75^ to generate mapped reads, which were converted to Bam format using Samtools for peak calling.^76^ The peaks were called using MACS2^77^ with the sonicated input as a control and *p*‐value of 0.01 as an initial cut‐off threshold. A consensus set of peaks were combined using irreproducible discovery rate^78^ after calling from each individual dataset with MACS2, and the conservative peak set was selected. *De novo* motifs from the C/EBPδ ChIP‐seq peaks were identified with Homer2.^45^ The genomic C/EBPδ binding sites were annotated using the ‘‘annotatePeaks.pl’’ script supplied by Homer2 as well. The following partitions were applied for observation of peak distribution features: promoter‐TSS (−1 kb ∼ +1 kb), transcription termination sites, exons, 5′UTR, 3′UTR, introns, non‐coding, and intergenic. Deeptools^79^ were used to calculate peak values and for visualization of read counts by converting raw bam files to bigwig files. The significant pathway of the genes with increased C/EBPδ binding on the promoter after cortisol treatment was analyzed according to KEGG database.^80^ Fisher's exact test was used to select the significant pathway, and the threshold of significance was defined by *p*‐value < 0.05.

### 4.10 ChIP assay to confirm the enrichment of C/EBPδ at *PTGS2* and *HSD11B1* promoters in amnion fibroblasts

ChIP assay was further performed on amnion fibroblasts to confirm the binding of C/EBPδ to *PTGS2* and *HSD11B1* promoters after treatment with (*n* = 4) and without (*n* = 4) cortisol (1 μM; 12 h). The method of ChIP is described as above. The primer sequences for qRT‐PCR are illustrated in Table S4, which amplified the promoter regions of −186 to −40 bp for *PTGS2* and −204 to −44 bp for *HSD11B1*, respectively. These regions overlap the peak spanning regions revealed by ChIP‐seq and contain the putative C/EBP binding site. The same amount of sheared DNA precipitated by the pre‐immune IgG or without antibody precipitation served as negative or input control, respectively. The ratio of DNA precipitated by C/EBPδ antibody over input control was calculated as a measure of bound C/EBPδ.

### 4.11 Mouse model to study distribution and gestational changes of C/EBPδ, COX‐2, and 11β‐HSD1 in the fetal membranes

Animal experimentation was conducted according to approved standards for animal care by the Institutional Review Board of Ren Ji Hospital, Shanghai Jiao Tong University School of Medicine. C57BL/6 mice (Charles River, Beijing, China) aging from 10 to 13 weeks were mated overnight. The presence of a vaginal plug in the morning was counted as gestational day 0.5. Fetal membranes were collected on gestational days 14.5 and 18.5, and fixed for the study of the distribution of C/EBPδ, COX‐2, and 11β‐HSD1 with immunohistochemical staining of paraffin‐embedded tissue sections using antibodies against C/EBPδ (GeneTex), COX‐2 (Cell Signaling Technology), and 11β‐HSD1 (Abcam). To study gestational changes of C/EBPδ, COX‐2, and 11β‐HSD1, fetal membranes were collected on gestational days 14.5, 15.5, 16.5, 17.5, and 18.5 for protein extraction and measurements of C/EBPδ, COX‐2, and 11β‐HSD1 abundance with Western blotting.

### 4.12 Construction of *Cebpd* knockout mice to study the role of C/EBPδ in parturition

*Cebpd* knockout mice were generated using a CRISPR/Cas9 system in Nanjing Biomedical Research Institute of Nanjing University, China. In the targeted mice, this CRISPR/Cas9‐mediated gene disruption led to a genomic deletion of the coding region of *Cebpd* (Figure S9A) with the pipeline illustrated in Figure S9B. Firstly, two sgRNAs targeting the both sides of the coding region of *Cebpd* were constructed respectively and transcribed *in vitro*. Cas9 mRNA and sgRNA were then co‐injected into the C57BL/6 zygotes. The sequences of sgRNA are illustrated in Figure S9C. Thereafter, the C57BL/6 zygotes were transferred into the oviduct of pseudo‐pregnant Institute of Cancer Reserach female mice at 0.5 dpc. All of the offspring was genotyped by PCR and sequencing using tail‐derived genomic DNA. The position and sequences of primers for PCR are illustrated in Figures S10A and S10B. Finally, after sexual maturity, the positive C57BL/6 mice were crossed with wild‐type C57BL/6 mice to build up heritable heterozygous mice (*Cebpd* ^+/−^). In order to observe the effect of the fraction of homozygous (*Cebpd* ^−/−^) embryos on the delivery time, sexually mature heterozygous (*Cebpd* ^+/−^) siblings were mated with each other. The delivery time was recorded, and all the offspring were genotyped by PCR and sequencing using tail‐derived genomic DNA. Representative gel images for identification of different genotypes were shown in Figure S10B. The fraction of homozygotes (*Cebpd* ^−/−^) in each pregnant mouse was counted, and the relationship between the fraction of homozygous embryos and delivery time was analyzed. To investigate whether *Cebpd* knock‐out affects the expression of COX‐2 and 11β‐HSD1, the abundance of COX‐2 and 11β‐HSD1 in the fetal membranes was compared between wild‐type and homozygous (*Cebpd* ^−/−^) embryos with Western blotting.

### 4.13 Statistical analysis

All data are presented as means ± SEM. The number of repeated experiments in each study was independent experiments reflecting independent patients. After normality testing with the Shapiro‐Wilk test, paired or unpaired Student's *t* test or Mann‐Whitney test or one‐way ANOVA test followed by the Newman‐Keuls Multiple Comparison Test was performed where appropriate. Statistical significance was defined as *p* < 0.05.

## AUTHOR CONTRIBUTIONS

Jiang‐Wen Lu, Wang‐Sheng Wang, and Kang Sun designed the project. Jiang‐Wen Lu and Wang‐Sheng Wang performed experiments. Jiang‐Wen Lu, Qiong Zhou, Li‐jun Ling, Hao Ying, and Yun Sun collected samples from patients and analyzed clinical information. Jiang‐Wen Lu, Wang‐Sheng Wang, Leslie Myatt, and Kang Sun analyzed the data and wrote the manuscript.

## CONFLICT OF INTEREST

The authors declare that they have no competing interests.

## Supporting information



Supporting Information

Supporting Information

Supporting Information

Supporting Information

Supporting Information

## Data Availability

RNA‐sequence data from amnion fibroblasts with or without cortisol treatment are available from the NCBI GEO accession GSE166320. RNA‐sequence data from the amnion tissue obtained from TL and TNL are available from the NCBI GEO accession GSE166453. ChIP‐sequence data from amnion fibroblasts with or without cortisol treatment are available from the NCBI GEO accession GSE166454.
