# Supplementary material for: C/EBPδ drives key endocrine signals in the human amnion at parturition
Source: Clin Transl Med. 2021 Jun 10;11(6):e416. doi: 10.1002/ctm2.416 (PMC8191398; doi:10.1002/ctm2.416)
Supplement: Supplementary file 1 — Supporting Information [file CTM2-11-e416-s003.docx]

**C/EBPδ drives key endocrine signals in the human amnion at parturition**

Jiang-wen Lu^a,b,1^, Wang-sheng Wang^a,b,1,^*, Qiong Zhou^c^, Li-jun Ling^d^, Hao Ying^d^, Yun Sun^a,b^, Leslie Myatt^e^, Kang Sun^a,b,^*

^a^Center for Reproductive Medicine, Ren Ji Hospital, School of Medicine, Shanghai Jiao Tong University. Shanghai, P.R. China.

^b^Shanghai Key Laboratory for Assisted Reproduction and Reproductive Genetics, Shanghai, P.R. China.

^c^Department of Obstetrics & Gynecology, Ren Ji Hospital, School of Medicine, Shanghai Jiao Tong University. Shanghai, P.R. China.

^d^Shanghai First Maternity and Infant Hospital, Tongji University School of Medicine, Shanghai, P.R. China.

^e^Department of Obstetrics and Gynecology, Oregon Health & Science University, Portland OR 97239, USA.

*To whom correspondence should be addressed: Kang Sun, M.D., Ph.D. or Wang-sheng Wang, Ph.D., Center for Reproductive Medicine, Ren Ji Hospital, School of Medicine, Shanghai Jiao Tong University, Shanghai 200135, P.R. China. E-mail: [sungangrenji@hotmail.com](mailto:sungangrenji@hotmail.com), [wangsheng_wang@hotmail.com](mailto:wangsheng_wang@hotmail.com).

^1^J.W.L. and W.S.W. contributed equally to this work.

**Contents**

Fig. S1. **mRNA abundance of C/EBP family members as revealed by the transcriptome study in human amnion tissue and fibroblasts**.

Fig. S2. **Effect of cortisol on *CEBPD* expression in human amnion epithelial cells.**

Fig. S3. **Effects of cortisol, PGE2 and IL-1β on *PTGS2* and *HSD11B1* expression in human amnion fibroblasts.**

Fig. S4. **Effects of cortisol, PGE2 and IL-1β on *CEBPA, CEBPB, CEBPG* and *DDIT3* expression in amnion fibroblasts.**

Fig. S5. **Effect of cortisol on *GAPDH* expression in human amnion fibroblasts.**

Fig. S6. **Effects of knock-down of C/EBPδ with another separate siRNA on the induction of *PTGS2* and *HSD11B1* expression by cortisol and PGE2 in human amnion fibroblasts.**

Fig. S7. **Litter size in normal term (19.5 days) and post-term (20.5 days) groups of mice.**

Fig. S8. **Western blot showing the specificity of the C/EBPδ antibody on the protein extracted from human amnion fibroblasts.**

Fig. S9. **Schematic diagram illustrating the construction of *Cebpd* knockout mice.**

Fig. S10. **Representative gel image showing the PCR products of different genotypes.**

Table S1. **Demographic and clinical characteristics of recruited pregnant subjects.**

Table S2. **Information on pregnancy, time of delivery and offspring genotypes in crosses between *Cebpd*^+/-^ mice.**

Table S3. **Information on antibodies used in this study.**

Table S4. **Primer sequences used in this study.**

Dataset 1. **Transcription factors with significant changes in the amnion tissue obtained following spontaneous labor and in amnion fibroblasts with cortisol treatment (1 μM; 24 hours).**

Dataset 2. **Peaks on chromosomes identified in amnion fibroblasts with and without cortisol treatment (1 μM; 12 hours) by ChIP-seq analysis.**

Dataset 3. **Genes with increased C/EBPδ enrichment on their promoters after cortisol treatment (1 μM; 12 hours) as revealed by ChIP-seq analysis.**


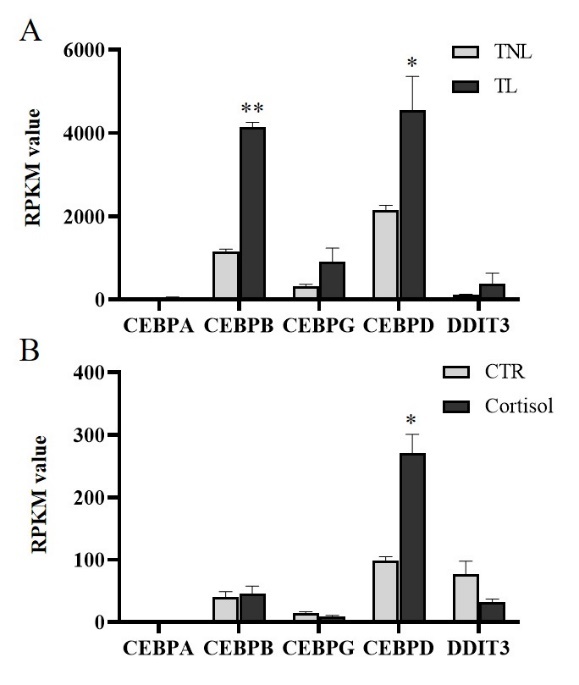


**Fig. S1.** **mRNA abundance of C/EBP family members as revealed by the transcriptome study in human amnion tissue and fibroblasts**. **(A)** RPKM values of *CEBPA* (C/EBPα), *CEBPB* (C/EBPβ), *CEBPG* (C/EBPγ), *CEBPD* (C/EBPδ) and *DDIT3* (C/EBPζ) mRNA in the human amnion tissue obtained from elective c section without labor at term (TNL, n=3) and spontaneous labor at term (TL, n=3). Data are means ± SEM, *P < 0.05, **P < 0.01 vs TNL (Unpaired Student’s t test). **(B)** RPKM values of *CEBPA* (C/EBPα), *CEBPB* (C/EBPβ), *CEBPG* (C/EBPγ), *CEBPD* (C/EBPδ) and *DDIT3* (C/EBPζ) mRNA in human amnion fibroblasts with or without cortisol treatment (1 μM; 24 hours, n=3). Data are means ± SEM, *P < 0.05 vs control without cortisol (Paired Student’s t test).


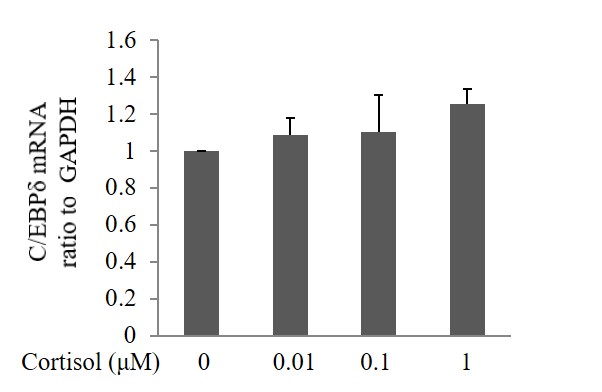


**Fig. S2. Effect of cortisol on *CEBPD* expression in human amnion epithelial cells.** There was no effect of cortisol (0.01, 0.1 and 1μM; 24 hours) on *CEBPD* mRNA abundance in human amnion epithelial cells. Data are means ± SEM from 4 experiments. P > 0.05 (one-way ANOVA followed by the Newman-Keuls multiple comparison test).


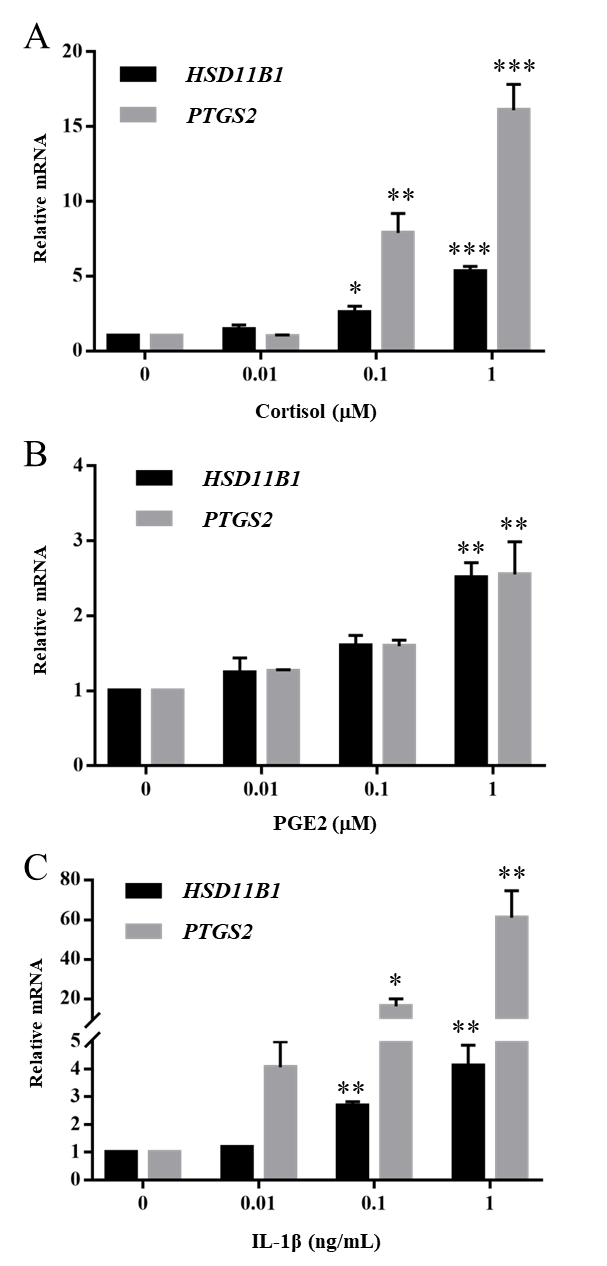


**Fig. S3. Effects of cortisol, PGE2 and IL-1β on *PTGS2* and *HSD11B1* expression in human amnion fibroblasts. (A**) Concentration-dependent effects of cortisol (0.01, 0.1 and 1μM; 24 hours); (**B**) Concentration-dependent effects of PGE2 (0.01, 0.1 and 1μM; 24 hours); (**C**) Concentration-dependent effects of IL-1β (0.01, 0.1 and 1 ng/mL; 24 hours). Data are means ± SEM from 3 experiments. *P < 0.05, **P < 0.01, ***P<0.001 vs untreated controls (one-way ANOVA followed by the Newman-Keuls multiple comparison test).


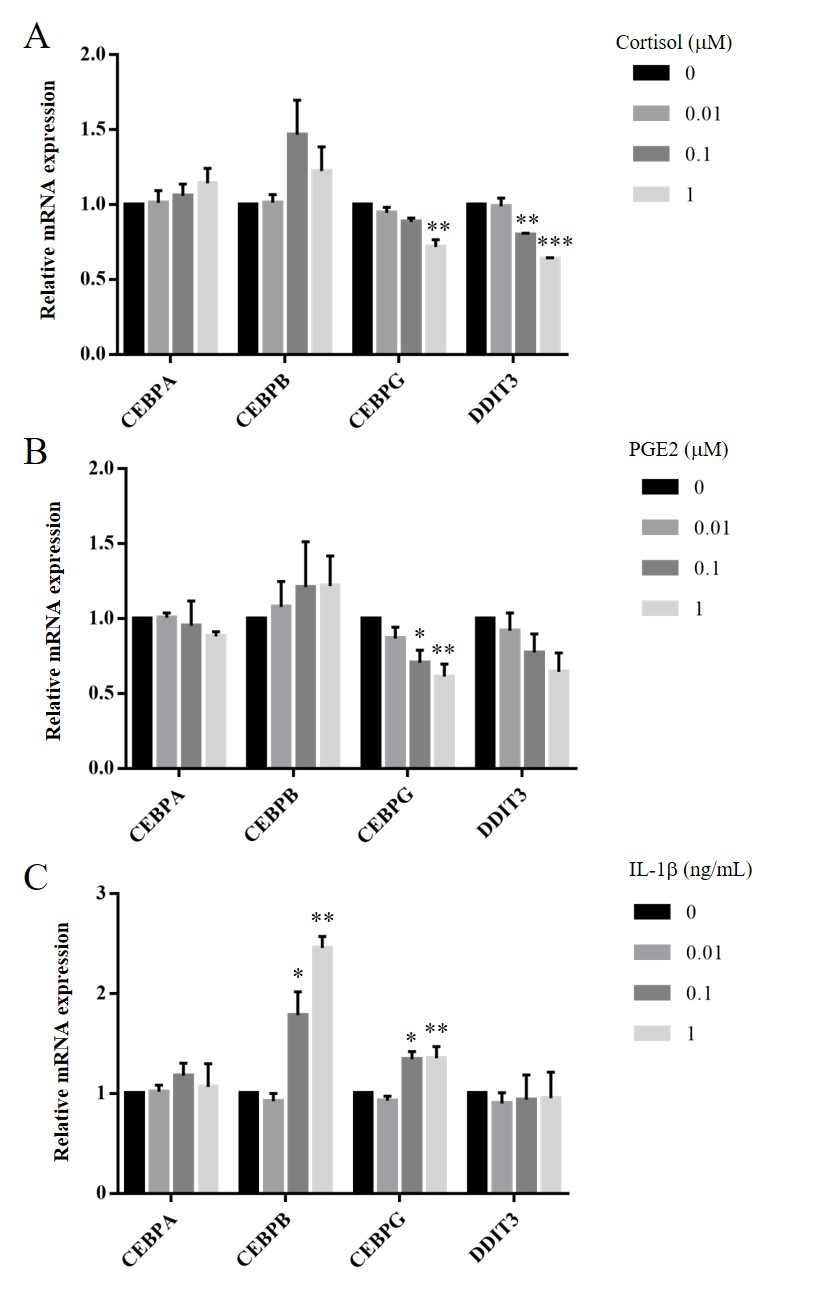


**Fig. S4. Effects of cortisol, PGE2 and IL-1β on *CEBPA, CEBPB, CEBPG* and *DDIT3* expression in amnion fibroblasts. (A)** Concentration-dependent effects of cortisol (0.01, 0.1 and 1μM; 24 hours); (**B**) Concentration-dependent effects of PGE2 (0.01, 0.1 and 1μM; 24 hours); (**C**) Concentration-dependent effects of IL-1β (0.01, 0.1 and 1 ng/mL; 24 hours). Data are means ± SEM from 3 experiments. *P < 0.05, **P < 0.01, ***P < 0.001 vs untreated controls (one-way ANOVA followed by the Newman-Keuls multiple comparison test).

**
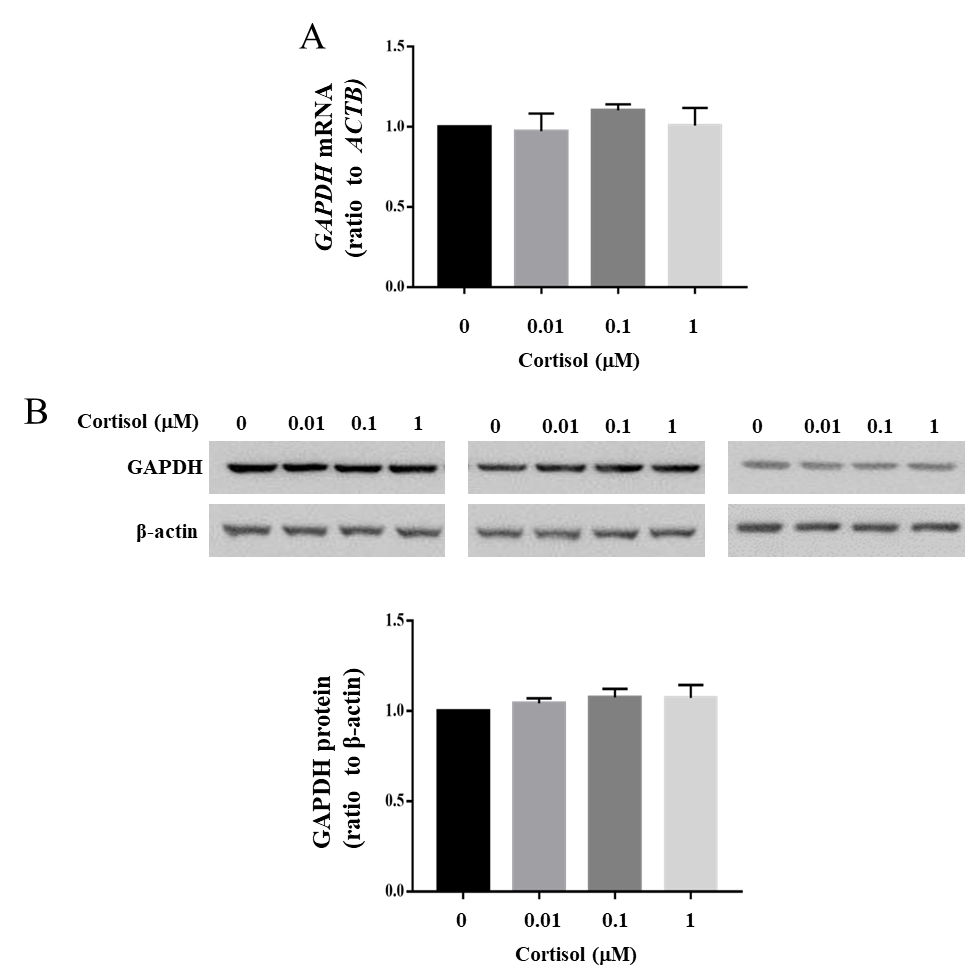
**

**Fig. S5. Effect of cortisol on *GAPDH* expression in human amnion fibroblasts.** There was no effect of cortisol (0.01, 0.1 and 1 μM; 24 hours) on either *GAPDH* mRNA (A) or protein abundance (B). Data are means ± SEM from 3 experiments, P>0.05 (one-way ANOVA followed by the Newman-Keuls multiple comparison test).


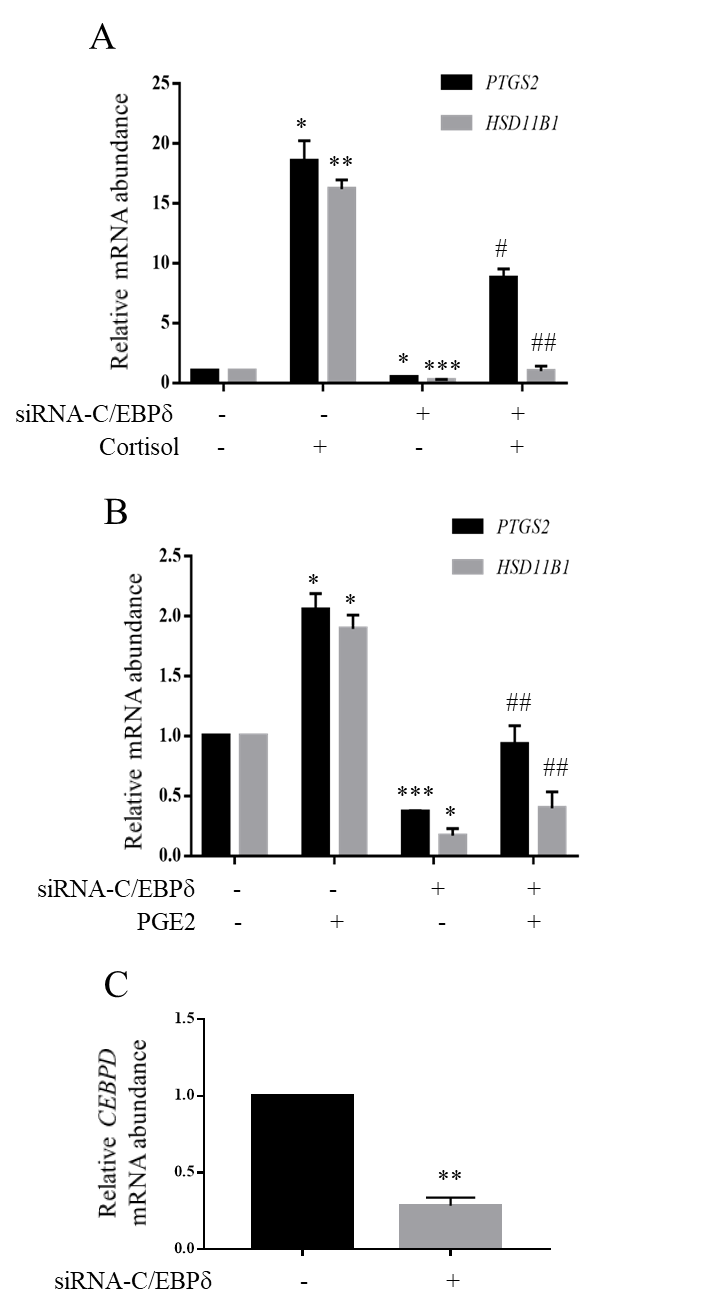


**Fig. S6. Effects of knock-down of C/EBPδ with another separate siRNA on the induction of *PTGS2* and *HSD11B1* expression by cortisol and PGE2 in human amnion fibroblasts. (A** and **B)** Quantification of *PTGS2* and *HSD11B1* mRNA in cortisol (1 μM; 24 hours; A) and PGE2 (1 μM; 24 hours; B)-treated human amnion fibroblasts transfected with scrambled (−) or C/EBPδ-targeted (+) siRNA. Data are mean ± SEM from 3 experiments. **(C)** The efficiency of siRNA-mediated knockdown of *CEBPD*. *P < 0.05, **P < 0.01, ***P < 0.001 against control with scrambled siRNA; #P < 0.05, ##P < 0.01 compared to cells treated with cortisol or PGE2 (A and B: One-way ANOVA followed by the Newman-Keuls multiple comparison test; C: Paired Student’s t test).


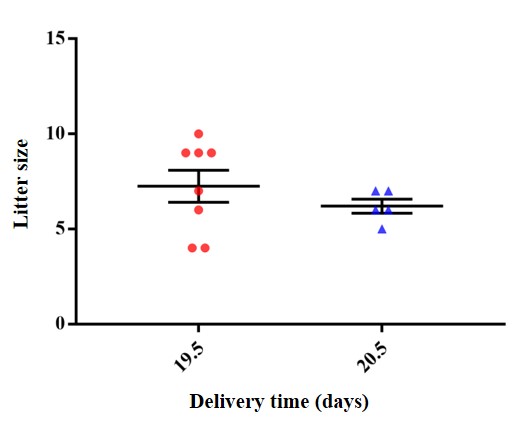


**Fig. S7. Litter size in normal term (19.5 days) and post-term (20.5 days) groups of mice.** Data are means ± SEM. P>0.05 (Unpaired Student’s t test).


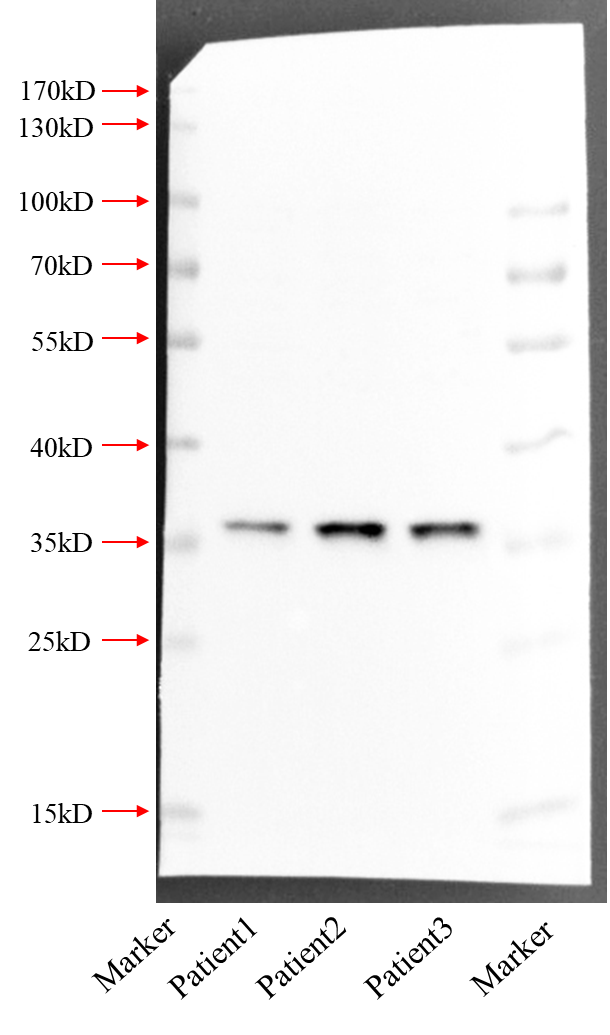


**Fig. S8. Western blot showing the specificity of the C/EBPδ antibody on the protein extracted from human amnion fibroblasts. Lanes 2, 3 and 4 represent isolates of amnion fibroblasts from independent patients.**

**
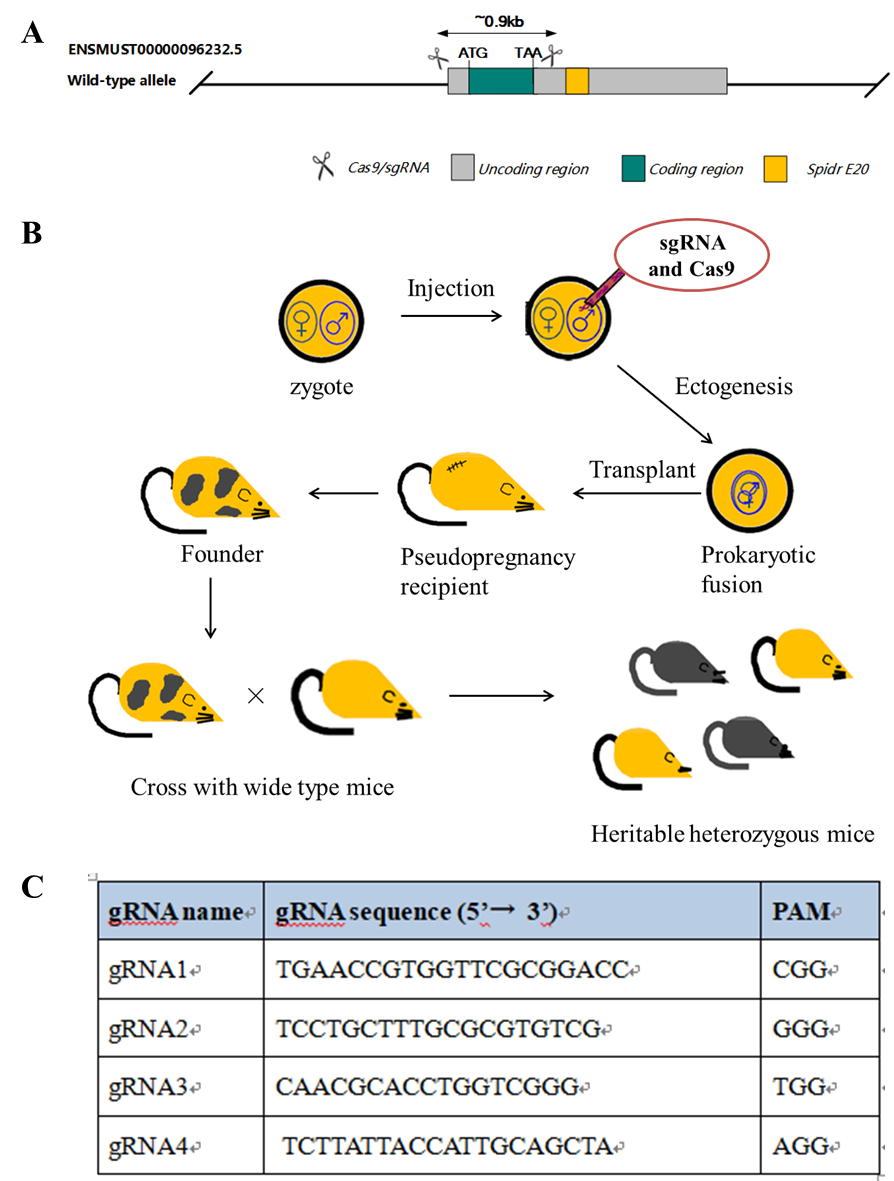
**

**Fig. S9. Schematic diagram illustrating the construction of *Cebpd* knockout mice.** **(A)** The diagram illustrates the position of CRISPR/Cas9-mediated gene disruption in the *Cebpd* gene. **(B)** The construction pipeline of *Cebpd* knockout mice. **(C)** sgRNA sequences. PAM, protospacer adjacent motif.

**
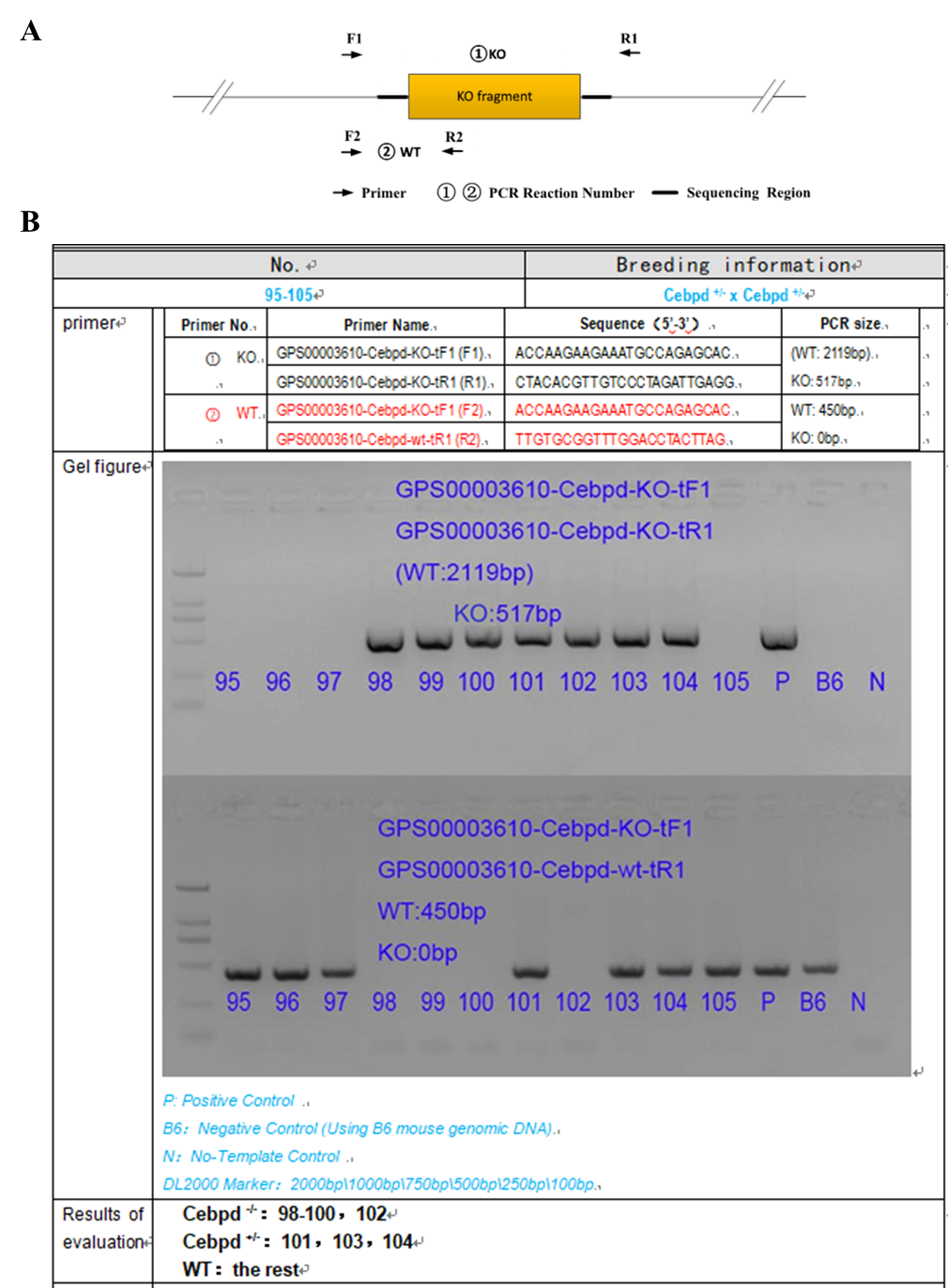
**

**Fig. S10. Representative gel image showing the PCR products of different genotypes. (A)** The aligning positions of PCR primers. **(B)** The primer sequences for PCR and the representative gel image of PCR products of different genotypes. WT, wide-type; KO, knockout.

| Demographic features | TNL | TL | P value |
| --- | --- | --- | --- |
| Maternal age (yr) | **31.3 ± 1.0** | **29.5 ± 0.5** | **0.14** |
| Body mass index (kg/m^2^) | **27.1 ± 1.6** | **25.2 ± 1.3** | **0.37** |
| Gravidity (median (range)) | **2（1-3）** | **2（1-4）** | **0.33** |
| Parity (median (range)) | **1（1-2）** | **1（1-2）** | **0.65** |
| Gestational age at delivery (wk) | **38.8 ± 0.2** | **39.2 ± 0.2** | **0.17** |
| Birth weight (g) | **3385 ± 138** | **3371 ± 128** | **0.94** |

**Table S1. Demographic and clinical characteristics of recruited pregnant subjects.**

1. Pregnant women undergoing spontaneous labor (TL) and elective c section without labor (TNL) at term
2. Pregnant women undergoing spontaneous labor (TL) and emergency c section with early

onset of labor (TL-CS) at term

| Demographic features | TNL | TL - CS | P value |
| --- | --- | --- | --- |
| Maternal age (yr) | **33.3 ± 0.7** | **31.3 ± 1.6** | **0.28** |
| Body mass index (kg/m^2^) | **27.6 ± 1.3** | **27.4 ± 2.2** | **0.92** |
| Gravidity (median (range)) | **2（1-4）** | **2（1-3）** | **0.37** |
| Parity (median (range)) | **2（1-3）** | **1（1-2）** | **0.21** |
| Gestational age at delivery (wk) | **39.0 ± 0.2** | **39.5 ± 0.2** | **0.13** |
| Birth weight (g) | **3376 ± 176** | **3221 ± 129** | **0.49** |

| Demographic features | A: 28-33W | B: 35-36W | C: 38W | P value | P value |
| --- | --- | --- | --- | --- | --- |
|  |  |  |  | **(A vs B)** | **(B vs C)** |
| Maternal age (yr) | **29.8 ± 2.1** | **34 ± 1.1** | **32.8 ± 1.3** | **0.128** | **0.504** |
| Body mass index (kg/m^2^) | **22.16 ± 1.1** | **25.3 ± 1.6** | **28.0 ± 1.5** | **0.170** | **0.256** |
| Gravidity (median (range)) | **1（1-3）** | **2.5（1-3）** | **2 (1-3)** | **0.476** | **0.714** |
| Parity (median (range)) | **1（1-2）** | **1（1-2）** | **1（1-2）** | **＞0.99** | **＞0.99** |
| Gestational age at delivery (wk) | **31.9 ± 0.8** | **36.0 ± 0.4** | **38.5 ± 0.1** | **0.004** | **0.005** |
| Birth weight (g) | **1597 ± 163** | **2568 ± 150** | **3442 ± 226** | **0.003** | **0.016** |

C. Pregnant women undergoing c section without labor at gestational ages ranging from 28 to 38 weeks

Parametric variables are expressed as mean ± SD and compared with unpaired Student’s t test. Nonparametric variables are expressed as median (min-max) and compared using Mann-Whitney U test. P＜0.05 was considered statistically significant.

**Table S2. Information on pregnancy, time of delivery and offspring genotypes in crosses between *Cebpd*^-/+^ mice.**

| **Parent No.** | **Parent genotype** | **Fetal No.** | **Fetal Gender** | **Birth date** | **Pregnant date** | **Offspring Genotype** | **Generation** | **condition** |
| --- | --- | --- | --- | --- | --- | --- | --- | --- |
| 6/101， | -1602bp/wt | 126 | ♂ | 2020/8/18 | 2020/7/30 | wt/wt | N2F1 | survival |
| 6/101， |  | 127 | ♂ | 2020/8/18 | 2020/7/30 | -1602bp/wt | N2F1 | survival |
| 6/101， |  | 128 | ♂ | 2020/8/18 | 2020/7/30 | -1602bp/-1602bp | N2F1 | survival |
| 6/101， |  | 129 | ♂ | 2020/8/18 | 2020/7/30 | -1602bp/-1602bp | N2F1 | survival |
| 6/101， |  | 130 | ♂ | 2020/8/18 | 2020/7/30 | -1602bp/wt | N2F1 | survival |
| 6/101， |  | 131 | ♂ | 2020/8/18 | 2020/7/30 | wt/wt | N2F1 | survival |
| 6/101， |  | 132 | ♂ | 2020/8/18 | 2020/7/30 | wt/wt | N2F1 | survival |
| 6/101， |  | 133 | ♂ | 2020/8/18 | 2020/7/30 | wt/wt | N2F1 | survival |
| 6/101， |  | 134 | ♀ | 2020/8/18 | 2020/7/30 | -1602bp/wt | N2F1 | survival |
|  |  |  |  |  |  |  |  |  |
| 2/84， | -1602bp/wt | 135 | ♂ | 2020/8/23 | 2020/8/4 | -1602bp/wt | N2F1 | survival |
| 2/84， |  | 136 | ♀ | 2020/8/23 | 2020/8/4 | -1602bp/wt | N2F1 | survival |
| 2/84， |  | 137 | ♀ | 2020/8/23 | 2020/8/4 | -1602bp/wt | N2F1 | survival |
| 2/84， |  | 138 | ♀ | 2020/8/23 | 2020/8/4 | wt/wt | N2F1 | survival |
|  |  |  |  |  |  |  |  |  |
| 9/31， | -1602bp/wt | 139 | ♂ | 2020/8/23 | 2020/8/4 | -1602bp/wt | N2F1 | survival |
| 9/31， |  | 140 | ♂ | 2020/8/23 | 2020/8/4 | -1602bp/wt | N2F1 | survival |
| 9/31， |  | 141 | ♂ | 2020/8/23 | 2020/8/4 | -1602bp/wt | N2F1 | survival |
| 9/31， |  | 142 | ♂ | 2020/8/23 | 2020/8/4 | -1602bp/wt | N2F1 | survival |
| 9/31， |  | 143 | ♀ | 2020/8/23 | 2020/8/4 | -1602bp/wt | N2F1 | survival |
| 9/31， |  | 144 | ♀ | 2020/8/23 | 2020/8/4 | -1602bp/wt | N2F1 | survival |
| 9/31， |  | 145 | ♀ | 2020/8/23 | 2020/8/4 | -1602bp/-1602bp | N2F1 | survival |
| 9/31， |  | 146 | ♀ | 2020/8/23 | 2020/8/4 | wt/wt | N2F1 | survival |
| 9/31， |  | 147 | ♀ | 2020/8/23 | 2020/8/4 | -1602bp/wt | N2F1 | survival |
|  |  |  |  |  |  |  |  |  |
| 29/50， | -1602bp/wt | 148 | ♂ | 2020/8/23 | 2020/8/4 | -1602bp/wt | N2F1 | survival |
| 29/50， |  | 149 | ♂ | 2020/8/23 | 2020/8/4 | -1602bp/wt | N2F1 | survival |
| 29/50， |  | 150 | ♂ | 2020/8/23 | 2020/8/4 | wt/wt | N2F1 | survival |
| 29/50， |  | 151 | ♂ | 2020/8/23 | 2020/8/4 | -1602bp/wt | N2F1 | survival |
| 29/50， |  | 152 | ♂ | 2020/8/23 | 2020/8/4 | -1602bp/-1602bp | N2F1 | survival |
| 29/50， |  | 153 | ♂ | 2020/8/23 | 2020/8/4 | -1602bp/wt | N2F1 | survival |
| 29/50， |  | 154 | ♂ | 2020/8/23 | 2020/8/4 | -1602bp/wt | N2F1 | survival |
| 29/50， |  | 155 | ♂ | 2020/8/23 | 2020/8/4 | -1602bp/-1602bp | N2F1 | survival |
| 29/50， |  | 156 | ♀ | 2020/8/23 | 2020/8/4 | -1602bp/wt | N2F1 | survival |
| 29/50， |  | 157 | ♂ | 2020/8/23 | 2020/8/4 | -1602bp/-1602bp | N2F1 | survival |
|  |  |  |  |  |  |  |  |  |
| 4/93， | -1602bp/wt | 158 | ♂ | 2020/8/23 | 2020/8/4 | wt/wt | N2F1 | survival |
| 4/93， |  | 159 | ♂ | 2020/8/23 | 2020/8/4 | -1602bp/-1602bp | N2F1 | survival |
| 4/93， |  | 160 | ♂ | 2020/8/23 | 2020/8/4 | -1602bp/wt | N2F1 | survival |
| 4/93， |  | 161 | ♂ | 2020/8/23 | 2020/8/4 | -1602bp/wt | N2F1 | survival |
|  |  |  |  |  |  |  |  |  |
| 10/32， | -1602bp/wt | 162 | ♂ | 2020/8/23 | 2020/8/4 | wt/wt | N2F1 | survival |
| 10/32， |  | 163 | ♂ | 2020/8/23 | 2020/8/4 | -1602bp/wt | N2F1 | survival |
| 10/32， |  | 164 | ♂ | 2020/8/23 | 2020/8/4 | wt/wt | N2F1 | survival |
| 10/32， |  | 165 | ♂ | 2020/8/23 | 2020/8/4 | wt/wt | N2F1 | survival |
| 10/32， |  | 166 | ♂ | 2020/8/23 | 2020/8/4 | wt/wt | N2F1 | survival |
| 10/32， |  | 167 | ♂ | 2020/8/23 | 2020/8/4 | -1602bp/wt | N2F1 | survival |
| 10/32， |  | 168 | ♀ | 2020/8/23 | 2020/8/4 | -1602bp/wt | N2F1 | survival |
| 10/32， |  | 169 | ♀ | 2020/8/23 | 2020/8/4 | -1602bp/wt | N2F1 | survival |
| 10/32， |  | 170 | ♀ | 2020/8/23 | 2020/8/4 | -1602bp/wt | N2F1 | survival |
|  |  |  |  |  |  |  |  |  |
| 4/24， | -1602bp/wt | 171 | ♂ | 2020/8/24 | 2020/8/4 | -1602bp/-1602bp | N2F1 | survival |
| 4/24， |  | 172 | ♂ | 2020/8/24 | 2020/8/4 | -1602bp/-1602bp | N2F1 | survival |
| 4/24， |  | 173 | ♂ | 2020/8/24 | 2020/8/4 | -1602bp/wt | N2F1 | survival |
| 4/24， |  | 174 | ♀ | 2020/8/24 | 2020/8/4 | wt/wt | N2F1 | survival |
| 4/24， |  | 175 | ♀ | 2020/8/24 | 2020/8/4 | -1602bp/-1602bp | N2F1 | survival |
| 4/24， |  | 176 | ♀ | 2020/8/24 | 2020/8/4 | -1602bp/wt | N2F1 | survival |
|  |  |  |  |  |  |  |  |  |
| 6/27， | -1602bp/wt | 177 | ♂ | 2020/8/24 | 2020/8/5 | -1602bp/wt | N2F1 | survival |
| 6/27， |  | 178 | ♂ | 2020/8/24 | 2020/8/5 | -1602bp/-1602bp | N2F1 | survival |
| 6/27， |  | 179 | ♂ | 2020/8/24 | 2020/8/5 | -1602bp/wt | N2F1 | survival |
| 6/27， |  | 180 | ♀ | 2020/8/24 | 2020/8/5 | -1602bp/wt | N2F1 | survival |
| 6/27， |  | 181 | ♀ | 2020/8/24 | 2020/8/5 | wt/wt | N2F1 | survival |
| 6/27， |  | 182 | ♀ | 2020/8/24 | 2020/8/5 | -1602bp/wt | N2F1 | survival |
|  |  |  |  |  |  |  |  |  |
| 3/15， | -1602bp/wt | 183 | ♂ | 2020/8/25 | 2020/8/5 | -1602bp/-1602bp | N2F1 | survival |
| 3/15， |  | 184 | ♂ | 2020/8/25 | 2020/8/5 | wt/wt | N2F1 | survival |
| 3/15， |  | 185 | ♂ | 2020/8/25 | 2020/8/5 | -1602bp/-1602bp | N2F1 | survival |
| 3/15， |  | 186 | ♂ | 2020/8/25 | 2020/8/5 | wt/wt | N2F1 | survival |
| 3/15， |  | 187 | ♀ | 2020/8/25 | 2020/8/5 | wt/wt | N2F1 | survival |
| 3/15， |  | 188 | ♀ | 2020/8/25 | 2020/8/5 | -1602bp/wt | N2F1 | survival |
| 3/15， |  | 189 | ♀ | 2020/8/25 | 2020/8/5 | wt/wt | N2F1 | survival |
|  |  |  |  |  |  |  |  |  |
| 9/103， | -1602bp/wt | 190 | ♂ | 2020/8/25 | 2020/8/5 | -1602bp/wt | N2F1 | survival |
| 9/103， |  | 191 | ♂ | 2020/8/25 | 2020/8/5 | -1602bp/-1602bp | N2F1 | survival |
| 9/103， |  | 192 | ♂ | 2020/8/25 | 2020/8/5 | -1602bp/-1602bp | N2F1 | survival |
| 9/103， |  | 193 | ♂ | 2020/8/25 | 2020/8/5 | wt/wt | N2F1 | survival |
| 9/103， |  | 194 | ♀ | 2020/8/25 | 2020/8/5 | wt/wt | N2F1 | survival |
| 9/103， |  | 195 | ♀ | 2020/8/25 | 2020/8/5 | -1602bp/-1602bp | N2F1 | survival |
|  |  |  |  |  |  |  |  |  |
| 5/94， | -1602bp/wt | 196 | ♂ | 2020/8/26 | 2020/8/7 | wt/wt | N2F1 | survival |
| 5/94， |  | 197 | ♀ | 2020/8/26 | 2020/8/7 | -1602bp/wt | N2F1 | survival |
| 5/94， |  | 198 | ♀ | 2020/8/26 | 2020/8/7 | -1602bp/-1602bp | N2F1 | survival |
| 5/94， |  | 199 | ♀ | 2020/8/26 | 2020/8/7 | wt/wt | N2F1 | survival |
| 5/94， |  | 200 | ♀ | 2020/8/26 | 2020/8/7 | -1602bp/wt | N2F1 | survival |
| 5/94， |  | 201 | ♀ | 2020/8/26 | 2020/8/7 | wt/wt | N2F1 | survival |
| 5/94， |  | 202 | ♀ | 2020/8/26 | 2020/8/7 | wt/wt | N2F1 | survival |
|  |  |  |  |  |  |  |  |  |
| 17/39， | -1602bp/wt | 203 | ♂ | 2020/8/26 | 2020/8/6 | -1602bp/wt | N2F1 | survival |
| 17/39， |  | 204 | ♂ | 2020/8/26 | 2020/8/6 | -1602bp/-1602bp | N2F1 | survival |
| 17/39， |  | 205 | ♂ | 2020/8/26 | 2020/8/6 | wt/wt | N2F1 | survival |
| 17/39， |  | 206 | ♂ | 2020/8/26 | 2020/8/6 | wt/wt | N2F1 | survival |
| 17/39， |  | 207 | ♂ | 2020/8/26 | 2020/8/6 | -1602bp/wt | N2F1 | survival |
| 17/39， |  | 208 | ♀ | 2020/8/26 | 2020/8/6 | wt/wt | N2F1 | survival |
| 17/39， |  | 209 | ♀ | 2020/8/26 | 2020/8/6 | -1602bp/-1602bp | N2F1 | survival |
|  |  |  |  |  |  |  |  |  |
| 28/43， | -1602bp/wt | 210 | ♂ | 2020/8/27 | 2020/8/7 | wt/wt | N2F1 | survival |
| 28/43， |  | 211 | ♂ | 2020/8/27 | 2020/8/7 | -1602bp/wt | N2F1 | survival |
| 28/43， |  | 212 | ♀ | 2020/8/27 | 2020/8/7 | -1602bp/wt | N2F1 | survival |
| 28/43， |  | 213 | ♀ | 2020/8/27 | 2020/8/7 | -1602bp/-1602bp | N2F1 | survival |
| 28/43， |  | 214 | ♀ | 2020/8/27 | 2020/8/7 | -1602bp/wt | N2F1 | survival |

| Antigen | Reactivity | Company | Catalog # | Dilution | | | |
| --- | --- | --- | --- | --- | --- | --- | --- |
|  |  |  |  | **WB** | **IHC** | **IF** | **ChIP** |
| CEBPδ | Human, Mouse | GeneTex | GTX115047 | 1:1000 | 1:100 | 1:50 | 1:50 |
| COX-2 | Human, Mouse | CST | 12282S | 1:2000 | 1:100 |  |  |
| 11β-HSD1 | Human | Abcam | ab157223 | 1:1000 |  |  |  |
| 11β-HSD1 | Mouse | Abcam | ab39364 | 1:2000 | 1:100 |  |  |
| GAPDH | Human, Mouse | Proteintech | 60004-1 | 1:10000 |  |  |  |
| Vimentin | Human | Santa Cruz | sc-6260 | 1:10000 |  | 1:200 |  |
| Lamin A/C | Human | CST | 4777S | 1:1000 |  |  |  |
| E-cadherin | Human | CST | 3195S | 1:1000 |  |  |  |
| β-actin | Human | Proteintech | 66009-1 | 1:10000 |  |  |  |

**Table S3. Information on antibodies used in this study**

WB, Western blotting; IHC, immunohistochemical staining; IF, immunofluorescence staining; ChIP, chromatin immunoprecipitation assay.

|  | Gene | Forward Primer (5’-3’) | | Reverse primer (5’-3’) |
| --- | --- | --- | --- | --- |
| qRT-PCR | CEBPD | | CATCGACTTCAGCGCCTACA | TTGAAGAGGTCGGCGAAGAG |
|  | HSD11B1 | | GCAGCCTCAGCACACTACAT | CATGTCTAGTCCTCCCATGAGC |
|  | PTGS2 | | TGTGCAACACTTGAGTGGCT | ACTTTCTGTACTGCGGGTG |
|  | GAPDH | | CCCCTCTGCTGATGCCCCCA | TGACCTTGGCCAGGGGTGCT |
|  | CEBPA | | TCGGTGGACAAGAACAGCAA | TTGTCACTGGTCAGCTCCAG |
|  | CEBPB | | AAGCACAGCGACGAGTACAA | ACAGCTGCTCCACCTTCTTC |
|  | CEBPG | | GGCTTACAGCAGGTTCCTCA | ATGTTGTTCCTCTCTCGGCG |
|  | CEBPE | | CAATCCCCTGCAGTACCAAGT | CCAAAGGGGCCTTGAGAACG |
|  | DDIT3 | | CCTGGAAATGAAGAGGAAGAATC | CTGACTGGAATCTGGAGAGTGA |
| ChIP | HSD11B1 | | CAGTCCTGTACAGTCATGAGCTTG | GTGCTAGCCAATTTCCCTGTCA |
|  | PTGS2 | | AGCTTCCTGGGTTTCCGATT | GCCCATGTGACGAAATGACTG |

**Table S4. Primer sequences used in this study.**

qRT-PCR, quantitative real time PCR; ChIP, chromatin immunoprecipitation assay.
