# Supplementary material for: C/EBPδ drives key endocrine signals in the human amnion at parturition
Source: Clin Transl Med. 2021 Jun 10;11(6):e416. doi: 10.1002/ctm2.416 (PMC8191398; doi:10.1002/ctm2.416)
Supplement: Supplementary file 2 — Supporting Information [file CTM2-11-e416-s005.pdf]

C/EBPδ drives key endocrine signals in the human amnion at parturition

Jiang-wen Lu[a,b,1], Wang-sheng Wang[a,b,1,*], Qiong Zhou[c], Li-jun Ling[d], Hao Ying[d], Yun Sun[a,b], Leslie Myatt[e], Kang Sun[a,b,*]

[a]Center for Reproductive Medicine, Ren Ji Hospital, School of Medicine, Shanghai Jiao Tong University. Shanghai, P.R. China.

[b]Shanghai Key Laboratory for Assisted Reproduction and Reproductive Genetics, Shanghai, P.R. China.

[c]Department of Obstetrics & Gynecology, Ren Ji Hospital, School of Medicine, Shanghai Jiao Tong University. Shanghai, P.R. China.

[d]Shanghai First Maternity and Infant Hospital, Tongji University School of Medicine, Shanghai, P.R. China.

[e]Department of Obstetrics and Gynecology, Oregon Health & Science University, Portland OR 97239, USA.

*To whom correspondence should be addressed: Kang Sun, M.D., Ph.D. or Wang-sheng Wang, Ph.D., Center for Reproductive Medicine, Ren Ji Hospital, School of Medicine, Shanghai Jiao Tong University, Shanghai 200135, P.R. China. E-mail: sungangrenji@hotmail.com, wangsheng_wang@hotmail.com.

[1]J.W.L. and W.S.W. contributed equally to this work.

Contents

Fig. S1. **mRNA abundance of C/EBP family members as revealed by the transcriptome study in human amnion tissue and fibroblasts**.

Fig. S2. **Effect of cortisol on *CEBPD* expression in human amnion epithelial cells.**

Fig. S3. **Effects of cortisol, PGE2 and IL-1β on *PTGS2* and *HSD11B1* expression in human amnion fibroblasts.**

Fig. S4. **Effects of cortisol, PGE2 and IL-1β on *CEBPA*, *CEBPB*, *CEBPG* and *DDIT3* expression in amnion fibroblasts.**

Fig. S5. **Effect of cortisol on *GAPDH* expression in human amnion fibroblasts.**

Fig. S6. **Effects of knock-down of C/EBPδ with another separate siRNA on the induction of *PTGS2* and *HSD11B1* expression by cortisol and PGE2 in human amnion fibroblasts.**

Fig. S7. **Litter size in normal term (19.5 days) and post-term (20.5 days) groups of mice.**

Fig. S8. **Western blot showing the specificity of the C/EBPδ antibody on the protein extracted from human amnion fibroblasts.**

Fig. S9. **Schematic diagram illustrating the construction of *Cebpd* knockout mice.**

Fig. S10. **Representative gel image showing the PCR products of different genotypes.**

Table S1. **Demographic and clinical characteristics of recruited pregnant subjects.**

Table S2. **Information on pregnancy, time of delivery and offspring genotypes in crosses between *Cebpd*$^{+/-}$ mice.**

Table S3. **Information on antibodies used in this study.**

Table S4. **Primer sequences used in this study.**

Dataset 1. **Transcription factors with significant changes in the amnion tissue obtained following spontaneous labor and in amnion fibroblasts with cortisol treatment (1 μM; 24 hours).**

Dataset 2. **Peaks on chromosomes identified in amnion fibroblasts with and without cortisol treatment (1 μM; 12 hours) by ChIP-seq analysis.**

Dataset 3. **Genes with increased C/EBPδ enrichment on their promoters after cortisol treatment (1 μM; 12 hours) as revealed by ChIP-seq analysis.**

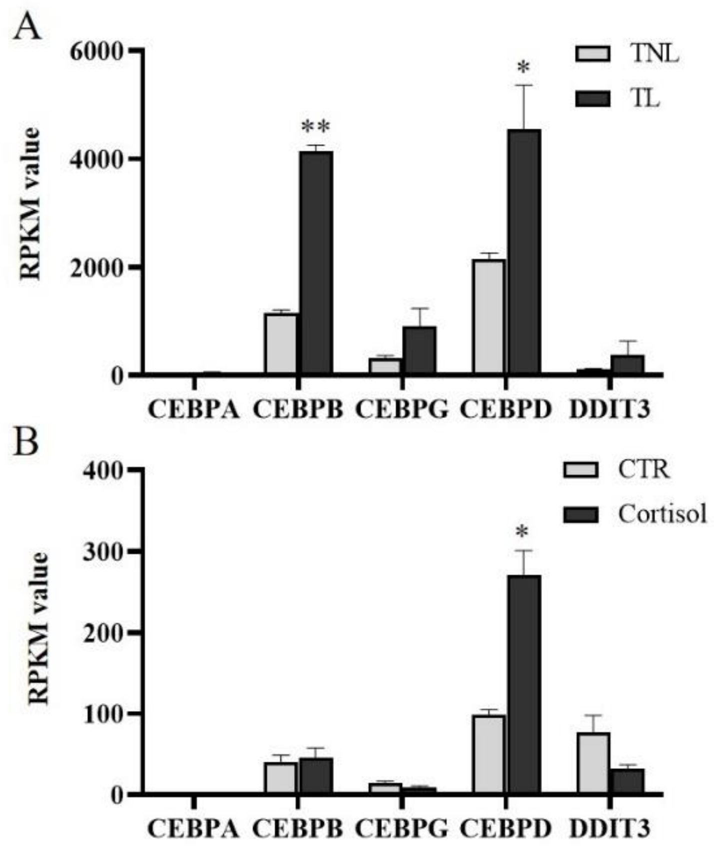

Fig. S1. mRNA abundance of C/EBP family members as revealed by the transcriptome study in human amnion tissue and fibroblasts. **(A)** RPKM values of *CEBPA* (C/EBPα), *CEBPB* (C/EBPβ), *CEBPG* (C/EBPγ), *CEBPD* (C/EBPδ) and *DDIT3* (C/EBPζ) mRNA in the human amnion tissue obtained from elective c section without labor at term (TNL, n=3) and spontaneous labor at term (TL, n=3). Data are means ± SEM, *P < 0.05, **P < 0.01 vs TNL (Unpaired Student's t test). **(B)** RPKM values of *CEBPA* (C/EBPα), *CEBPB* (C/EBPβ), *CEBPG* (C/EBPγ), *CEBPD* (C/EBPδ) and *DDIT3* (C/EBPζ) mRNA in human amnion fibroblasts with or without cortisol treatment (1 μM; 24 hours, n=3). Data are means ± SEM, *P < 0.05 vs control without cortisol (Paired Student's t test).

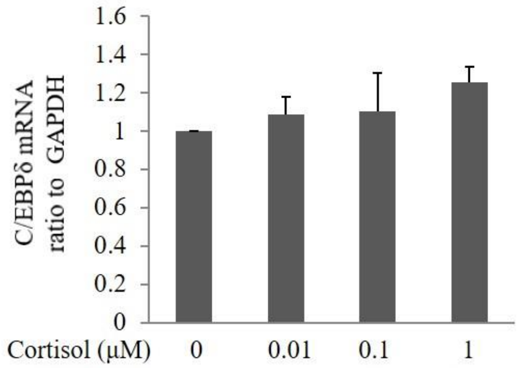

Fig. S2. Effect of cortisol on *CEBPD* expression in human amnion epithelial cells. There was no effect of cortisol (0.01, 0.1 and 1μM; 24 hours) on *CEBPD* mRNA abundance in human amnion epithelial cells. Data are means ± SEM from 4 experiments. $P > 0.05$ (one-way ANOVA followed by the Newman-Keuls multiple comparison test).

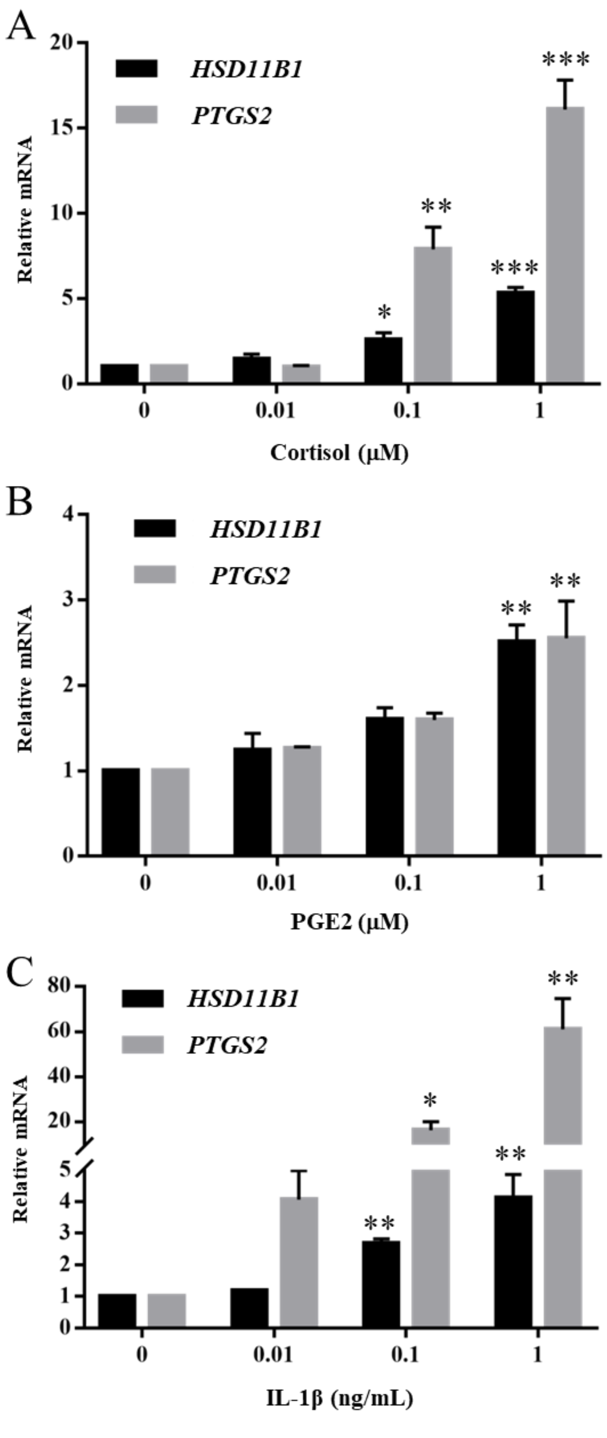

Fig. S3. Effects of cortisol, PGE2 and IL-1β on *PTGS2* and *HSD11B1* expression in human amnion fibroblasts. (A) Concentration-dependent effects of cortisol (0.01, 0.1 and 1μM; 24 hours); (**B**) Concentration-dependent effects of PGE2 (0.01, 0.1 and 1μM; 24 hours); (**C**) Concentration-dependent effects of IL-1β (0.01, 0.1 and 1 ng/mL; 24 hours). Data are means ± SEM from 3 experiments. *P < 0.05, **P < 0.01, ***P<0.001 vs untreated controls (one-way ANOVA followed by the Newman-Keuls multiple comparison test).

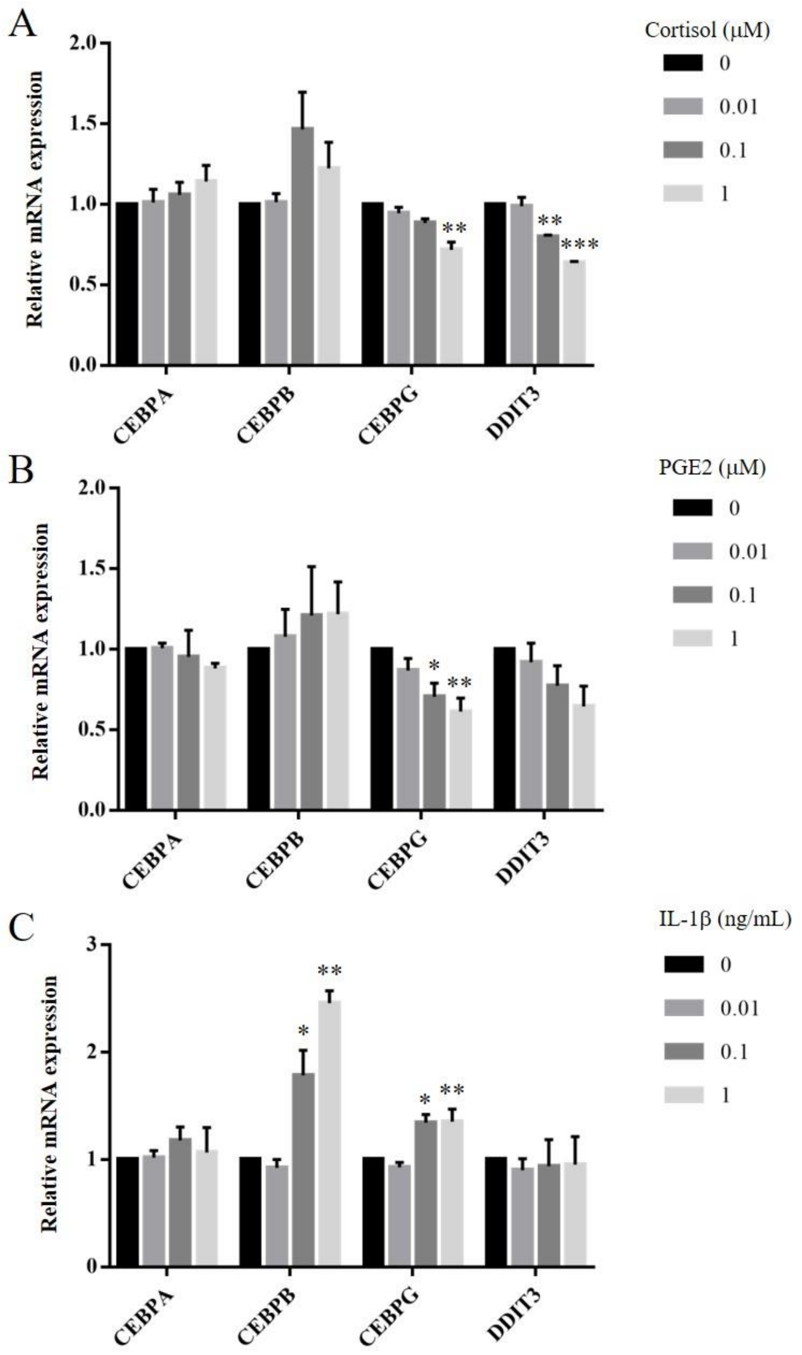

Fig. S4. Effects of cortisol, PGE2 and IL-1β on *CEBPA, CEBPB, CEBPG* and *DDIT3* expression in amnion fibroblasts. (A) Concentration-dependent effects of cortisol (0.01, 0.1 and 1μM; 24 hours); (**B**) Concentration-dependent effects of PGE2 (0.01, 0.1 and 1μM; 24 hours); (**C**) Concentration-dependent effects of IL-1β (0.01, 0.1 and 1 ng/mL; 24 hours). Data are means ± SEM from 3 experiments. *P < 0.05, **P < 0.01, ***P < 0.001 vs untreated controls (one-way ANOVA followed by the Newman-Keuls multiple comparison test).

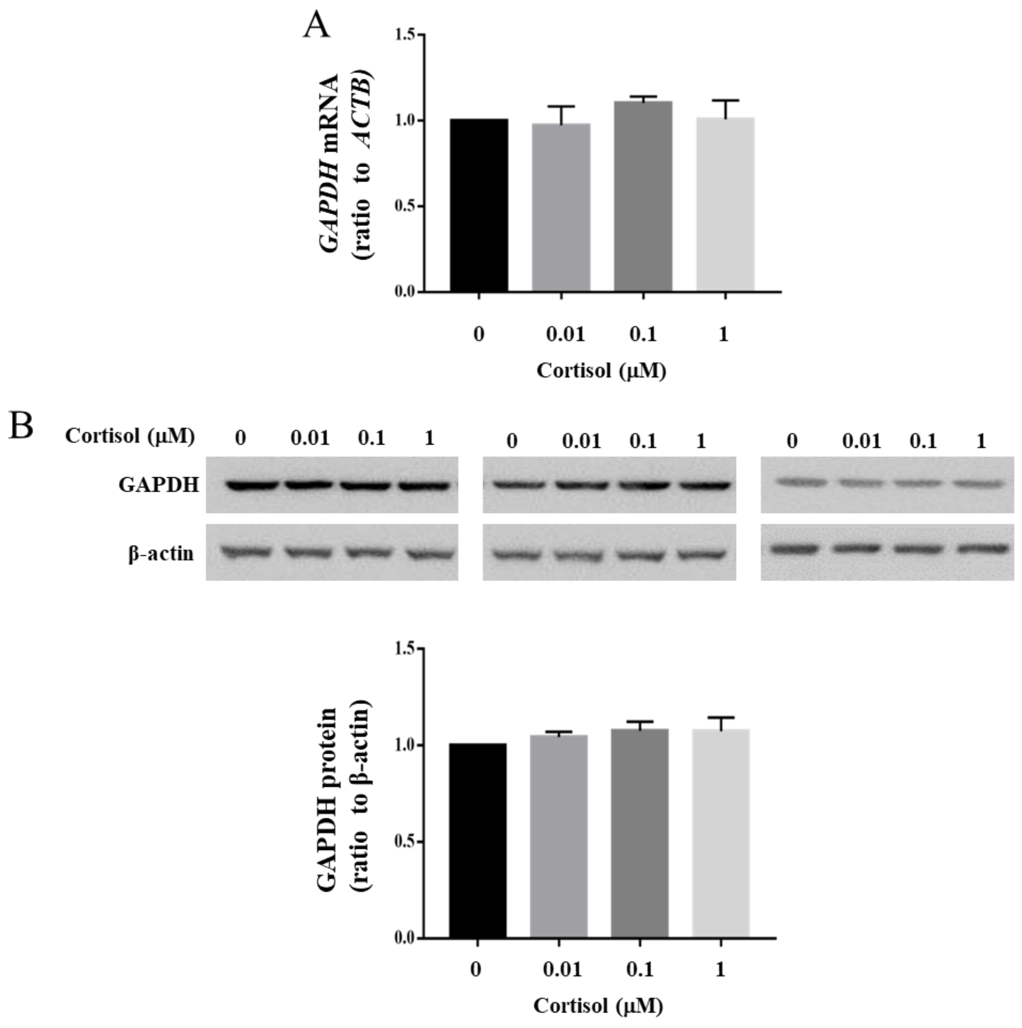

Fig. S5. Effect of cortisol on *GAPDH* expression in human amnion fibroblasts. There was no effect of cortisol (0.01, 0.1 and 1 μM; 24 hours) on either *GAPDH* mRNA (A) or protein abundance (B). Data are means ± SEM from 3 experiments, $P>0.05$ (one-way ANOVA followed by the Newman-Keuls multiple comparison test).

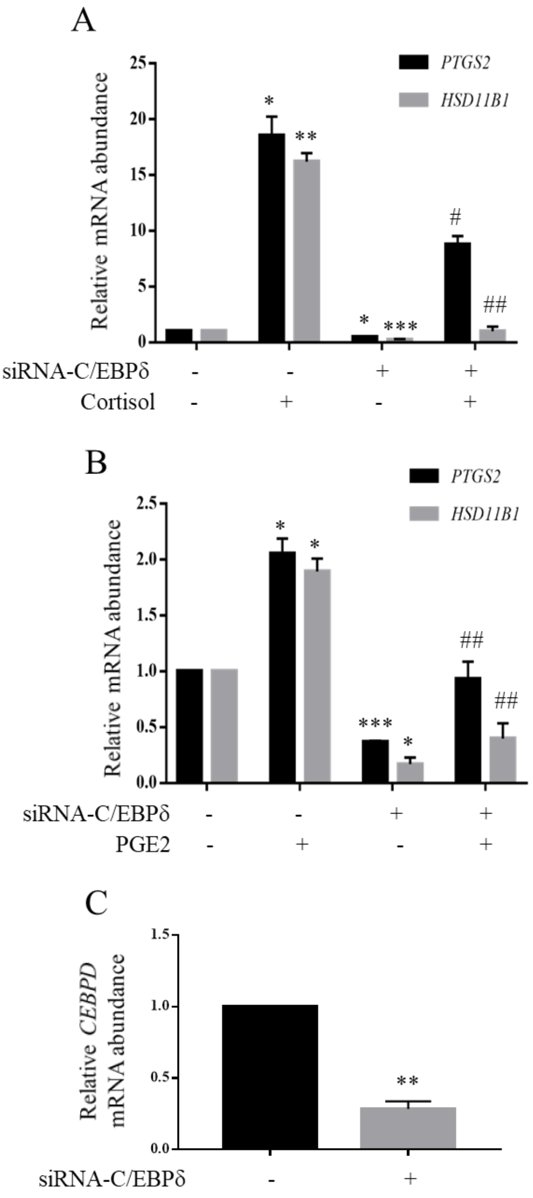

Fig. S6. Effects of knock-down of C/EBPδ with another separate siRNA on the induction of *PTGS2* and *HSD11B1* expression by cortisol and PGE2 in human amnion fibroblasts. (A and **B)** Quantification of *PTGS2* and *HSD11B1* mRNA in cortisol (1 μM; 24 hours; A) and PGE2 (1 μM; 24 hours; B)-treated human amnion fibroblasts transfected with scrambled (−) or C/EBPδ-targeted (+) siRNA. Data are mean ± SEM from 3 experiments. **(C)** The efficiency of siRNA-mediated knockdown of *CEBPD*. *$P < 0.05$, **$P < 0.01$, ***$P < 0.001$ against control with scrambled siRNA; #$P < 0.05$, ##$P < 0.01$ compared to cells treated with cortisol or PGE2 (A and B: One-way ANOVA followed by the Newman-Keuls multiple comparison test; C: Paired Student's t test).

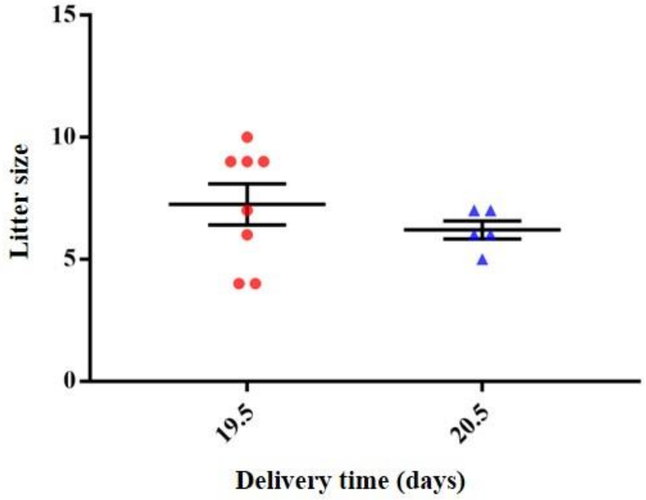

Fig. S7. Litter size in normal term (19.5 days) and post-term (20.5 days) groups of mice. Data are means ± SEM. P>0.05 (Unpaired Student's t test).

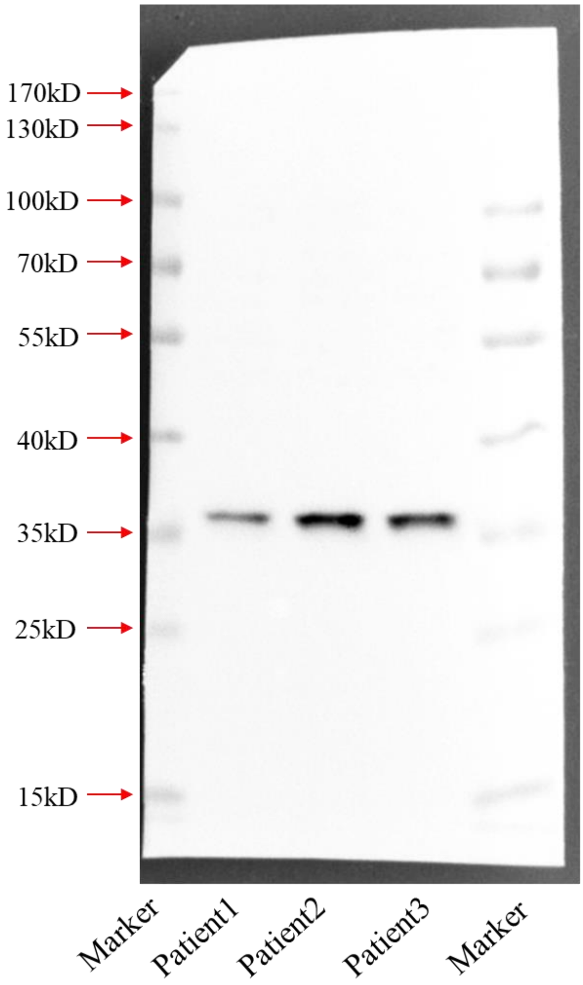

Fig. S8. Western blot showing the specificity of the C/EBPδ antibody on the protein extracted from human amnion fibroblasts. Lanes 2, 3 and 4 represent isolates of amnion fibroblasts from independent patients.

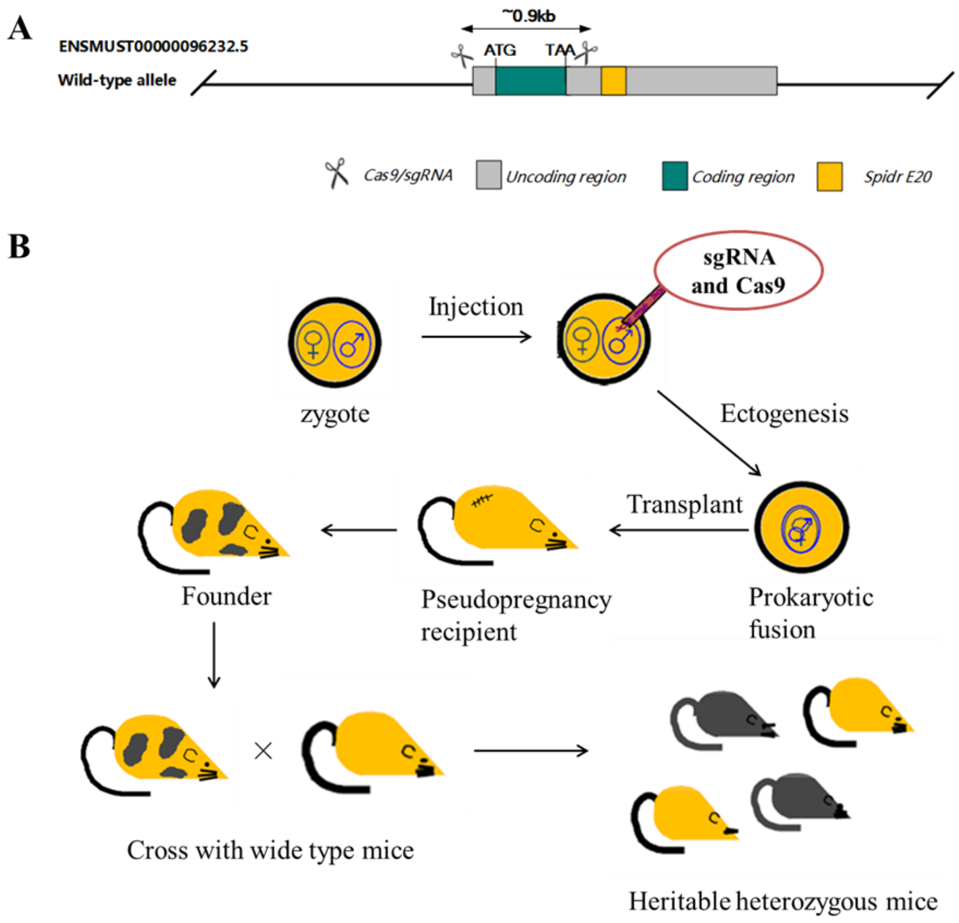

C

gRNA name	gRNA sequence (5'→ 3')	PAM
gRNA1	TGAACCGTGGTTCGCGGACC	CGG
gRNA2	TCCTGCTTTGCGCGTGTCG	GGG
gRNA3	CAACGCACCTGGTCGGG	TGG
gRNA4	TCTTATTACCATTGCAGCTA	AGG

Fig. S9. Schematic diagram illustrating the construction of *Cebpd* knockout mice. (A) The diagram illustrates the position of CRISPR/Cas9-mediated gene disruption in the *Cebpd* gene. **(B)** The construction pipeline of *Cebpd* knockout mice. **(C)** sgRNA sequences. PAM, protospacer adjacent motif.

A

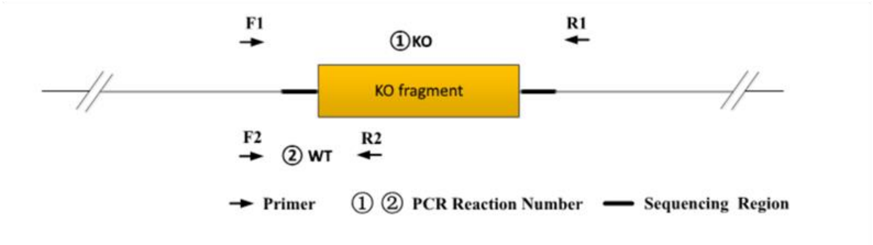

B

No.	Breeding information
95-105	Cebpd $^{+/-}$ x Cebpd $^{+/-}$

primer	Primer No.	Primer Name	Sequence (5'-3')	PCR size
	① KO	GPS00003610-Cebpd-KO-tF1 (F1)	ACCAAGAAGAAATGCCAGAGCAC	(WT: 2119bp)
		GPS00003610-Cebpd-KO-tR1 (R1)	CTACACGTTGTCCCTAGATTGAGG	KO: 517bp
	② WT	GPS00003610-Cebpd-KO-tF1 (F2)	ACCAAGAAGAAATGCCAGAGCAC	WT: 450bp
		GPS00003610-Cebpd-wt-tR1 (R2)	TTGTGCGGTTTGGACCTACTTAG	KO: 0bp

Gel figure

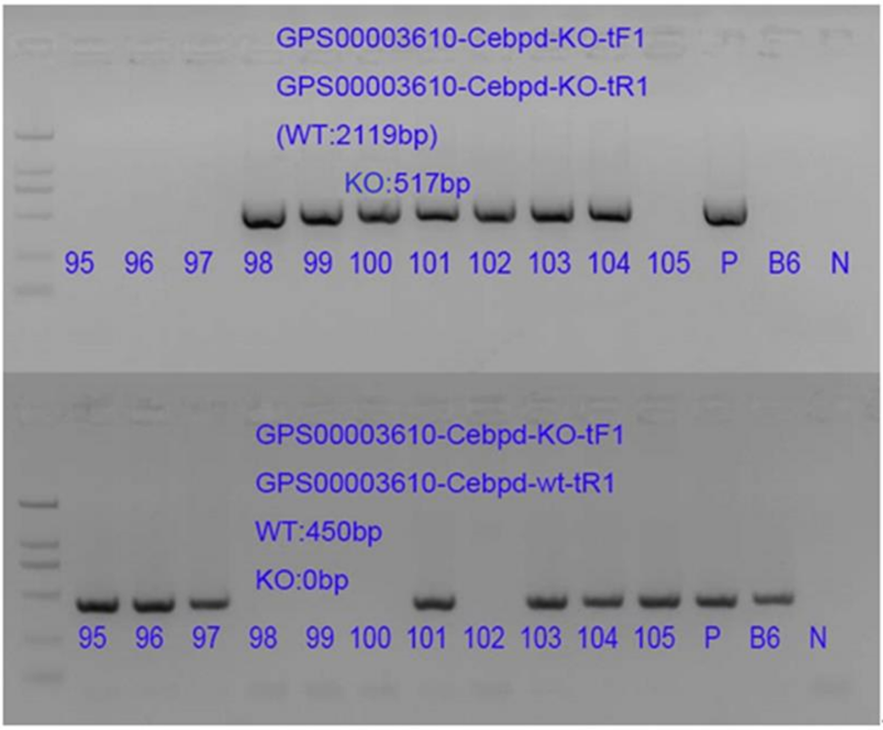

P: Positive Control

B6: Negative Control (Using B6 mouse genomic DNA).

N: No-Template Control

DL2000 Marker: 2000bp\1000bp\750bp\500bp\250bp\100bp.

Results of evaluation

Cebpd $^{-/-}$: 98-100, 102

Cebpd $^{+/-}$: 101, 103, 104

WT: the rest

Fig. S10. Representative gel image showing the PCR products of different genotypes. (A) The aligning positions of PCR primers. **(B)** The primer sequences for PCR and the representative gel image of PCR products of different genotypes. WT, wide-type; KO, knockout.

Table S1. Demographic and clinical characteristics of recruited pregnant subjects.

A. Pregnant women undergoing spontaneous labor (TL) and elective c section without labor (TNL) at term

Demographic features	TNL	TL	P value
Maternal age (yr)	31.3 ± 1.0	29.5 ± 0.5	0.14
Body mass index (kg/m^2)	27.1 ± 1.6	25.2 ± 1.3	0.37
Gravidity (median (range))	2 (1-3)	2 (1-4)	0.33
Parity (median (range))	1 (1-2)	1 (1-2)	0.65
Gestational age at delivery (wk)	38.8 ± 0.2	39.2 ± 0.2	0.17
Birth weight (g)	3385 ± 138	3371 ± 128	0.94

B. Pregnant women undergoing spontaneous labor (TL) and emergency c section with early onset of labor (TL-CS) at term

Demographic features	TNL	TL - CS	P value
Maternal age (yr)	33.3 ± 0.7	31.3 ± 1.6	0.28
Body mass index (kg/m^2)	27.6 ± 1.3	27.4 ± 2.2	0.92
Gravidity (median (range))	2 (1-4)	2 (1-3)	0.37
Parity (median (range))	2 (1-3)	1 (1-2)	0.21
Gestational age at delivery (wk)	39.0 ± 0.2	39.5 ± 0.2	0.13
Birth weight (g)	3376 ± 176	3221 ± 129	0.49

C. Pregnant women undergoing c section without labor at gestational ages ranging from 28 to 38 weeks

Demographic features	A: 28-33W	B: 35-36W	C: 38W	P value (A vs B)	P value (B vs C)
Maternal age (yr)	29.8 ± 2.1	34 ± 1.1	32.8 ± 1.3	0.128	0.504
Body mass index (kg/m^2)	22.16 ± 1.1	25.3 ± 1.6	28.0 ± 1.5	0.170	0.256
Gravidity (median (range))	1 (1-3)	2.5 (1-3)	2 (1-3)	0.476	0.714
Parity (median (range))	1 (1-2)	1 (1-2)	1 (1-2)	> 0.99	> 0.99
Gestational age at delivery (wk)	31.9 ± 0.8	36.0 ± 0.4	38.5 ± 0.1	0.004	0.005
Birth weight (g)	1597 ± 163	2568 ± 150	3442 ± 226	0.003	0.016

Parametric variables are expressed as mean ± SD and compared with unpaired Student's t test. Nonparametric variables are expressed as median (min-max) and compared using Mann-Whitney U test. $P < 0.05$ was considered statistically significant.

Table S2. Information on pregnancy, time of delivery and offspring genotypes in crosses between *Cebpd*$^{-/+}$ mice.

Parent No.	Parent genotype	Fetal No.	Fetal Gender	Birth date	Pregnant date	Offspring Genotype	Generation	condition
6/101,	−1602bp/wt	126	♂	2020/8/18	2020/7/30	wt/wt	N2F1	survival
6/101,		127	♂	2020/8/18	2020/7/30	−1602bp/wt	N2F1	survival
6/101,		128	♂	2020/8/18	2020/7/30	−1602bp/−1602bp	N2F1	survival
6/101,		129	♂	2020/8/18	2020/7/30	−1602bp/−1602bp	N2F1	survival
6/101,		130	♂	2020/8/18	2020/7/30	−1602bp/wt	N2F1	survival
6/101,		131	♂	2020/8/18	2020/7/30	wt/wt	N2F1	survival
6/101,		132	♂	2020/8/18	2020/7/30	wt/wt	N2F1	survival
6/101,		133	♂	2020/8/18	2020/7/30	wt/wt	N2F1	survival
6/101,		134	♀	2020/8/18	2020/7/30	−1602bp/wt	N2F1	survival
2/84,	−1602bp/wt	135	♂	2020/8/23	2020/8/4	−1602bp/wt	N2F1	survival
2/84,		136	♀	2020/8/23	2020/8/4	−1602bp/wt	N2F1	survival
2/84,		137	♀	2020/8/23	2020/8/4	−1602bp/wt	N2F1	survival
2/84,		138	♀	2020/8/23	2020/8/4	wt/wt	N2F1	survival
9/31,	−1602bp/wt	139	♂	2020/8/23	2020/8/4	−1602bp/wt	N2F1	survival
9/31,		140	♂	2020/8/23	2020/8/4	−1602bp/wt	N2F1	survival
9/31,		141	♂	2020/8/23	2020/8/4	−1602bp/wt	N2F1	survival
9/31,		142	♂	2020/8/23	2020/8/4	−1602bp/wt	N2F1	survival
9/31,		143	♀	2020/8/23	2020/8/4	−1602bp/wt	N2F1	survival
9/31,		144	♀	2020/8/23	2020/8/4	−1602bp/wt	N2F1	survival
9/31,		145	♀	2020/8/23	2020/8/4	−1602bp/−1602bp	N2F1	survival
9/31,		146	♀	2020/8/23	2020/8/4	wt/wt	N2F1	survival
9/31,		147	♀	2020/8/23	2020/8/4	−1602bp/wt	N2F1	survival
29/50,	−1602bp/wt	148	♂	2020/8/23	2020/8/4	−1602bp/wt	N2F1	survival
29/50,		149	♂	2020/8/23	2020/8/4	−1602bp/wt	N2F1	survival
29/50,		150	♂	2020/8/23	2020/8/4	wt/wt	N2F1	survival
29/50,		151	♂	2020/8/23	2020/8/4	−1602bp/wt	N2F1	survival
29/50,		152	♂	2020/8/23	2020/8/4	−1602bp/−1602bp	N2F1	survival
29/50,		153	♂	2020/8/23	2020/8/4	−1602bp/wt	N2F1	survival
29/50,		154	♂	2020/8/23	2020/8/4	−1602bp/wt	N2F1	survival
29/50,		155	♂	2020/8/23	2020/8/4	−1602bp/−1602bp	N2F1	survival
29/50,		156	♀	2020/8/23	2020/8/4	−1602bp/wt	N2F1	survival
29/50,		157	♂	2020/8/23	2020/8/4	−1602bp/−1602bp	N2F1	survival
4/93,	−1602bp/wt	158	♂	2020/8/23	2020/8/4	wt/wt	N2F1	survival
4/93,		159	♂	2020/8/23	2020/8/4	−1602bp/−1602bp	N2F1	survival

4/93,		160	♂	2020/8/23	2020/8/4	-1602bp/wt	N2F1	survival
4/93,		161	♂	2020/8/23	2020/8/4	-1602bp/wt	N2F1	survival
10/32,	-1602bp/wt	162	♂	2020/8/23	2020/8/4	wt/wt	N2F1	survival
10/32,		163	♂	2020/8/23	2020/8/4	-1602bp/wt	N2F1	survival
10/32,		164	♂	2020/8/23	2020/8/4	wt/wt	N2F1	survival
10/32,		165	♂	2020/8/23	2020/8/4	wt/wt	N2F1	survival
10/32,		166	♂	2020/8/23	2020/8/4	wt/wt	N2F1	survival
10/32,		167	♂	2020/8/23	2020/8/4	-1602bp/wt	N2F1	survival
10/32,		168	♀	2020/8/23	2020/8/4	-1602bp/wt	N2F1	survival
10/32,		169	♀	2020/8/23	2020/8/4	-1602bp/wt	N2F1	survival
10/32,		170	♀	2020/8/23	2020/8/4	-1602bp/wt	N2F1	survival
4/24,	-1602bp/wt	171	♂	2020/8/24	2020/8/4	-1602bp/-1602bp	N2F1	survival
4/24,		172	♂	2020/8/24	2020/8/4	-1602bp/-1602bp	N2F1	survival
4/24,		173	♂	2020/8/24	2020/8/4	-1602bp/wt	N2F1	survival
4/24,		174	♀	2020/8/24	2020/8/4	wt/wt	N2F1	survival
4/24,		175	♀	2020/8/24	2020/8/4	-1602bp/-1602bp	N2F1	survival
4/24,		176	♀	2020/8/24	2020/8/4	-1602bp/wt	N2F1	survival
6/27,	-1602bp/wt	177	♂	2020/8/24	2020/8/5	-1602bp/wt	N2F1	survival
6/27,		178	♂	2020/8/24	2020/8/5	-1602bp/-1602bp	N2F1	survival
6/27,		179	♂	2020/8/24	2020/8/5	-1602bp/wt	N2F1	survival
6/27,		180	♀	2020/8/24	2020/8/5	-1602bp/wt	N2F1	survival
6/27,		181	♀	2020/8/24	2020/8/5	wt/wt	N2F1	survival
6/27,		182	♀	2020/8/24	2020/8/5	-1602bp/wt	N2F1	survival
3/15,	-1602bp/wt	183	♂	2020/8/25	2020/8/5	-1602bp/-1602bp	N2F1	survival
3/15,		184	♂	2020/8/25	2020/8/5	wt/wt	N2F1	survival
3/15,		185	♂	2020/8/25	2020/8/5	-1602bp/-1602bp	N2F1	survival
3/15,		186	♂	2020/8/25	2020/8/5	wt/wt	N2F1	survival
3/15,		187	♀	2020/8/25	2020/8/5	wt/wt	N2F1	survival
3/15,		188	♀	2020/8/25	2020/8/5	-1602bp/wt	N2F1	survival
3/15,		189	♀	2020/8/25	2020/8/5	wt/wt	N2F1	survival
9/103,	-1602bp/wt	190	♂	2020/8/25	2020/8/5	-1602bp/wt	N2F1	survival
9/103,		191	♂	2020/8/25	2020/8/5	-1602bp/-1602bp	N2F1	survival
9/103,		192	♂	2020/8/25	2020/8/5	-1602bp/-1602bp	N2F1	survival
9/103,		193	♂	2020/8/25	2020/8/5	wt/wt	N2F1	survival
9/103,		194	♀	2020/8/25	2020/8/5	wt/wt	N2F1	survival
9/103,		195	♀	2020/8/25	2020/8/5	-1602bp/-1602bp	N2F1	survival
5/94,	-1602bp/wt	196	♂	2020/8/26	2020/8/7	wt/wt	N2F1	survival

5/94,		197	♀	2020/8/26	2020/8/7	-1602bp/wt	N2F1	survival
5/94,		198	♀	2020/8/26	2020/8/7	-1602bp/-1602bp	N2F1	survival
5/94,		199	♀	2020/8/26	2020/8/7	wt/wt	N2F1	survival
5/94,		200	♀	2020/8/26	2020/8/7	-1602bp/wt	N2F1	survival
5/94,		201	♀	2020/8/26	2020/8/7	wt/wt	N2F1	survival
5/94,		202	♀	2020/8/26	2020/8/7	wt/wt	N2F1	survival
17/39,	-1602bp/wt	203	♂	2020/8/26	2020/8/6	-1602bp/wt	N2F1	survival
17/39,		204	♂	2020/8/26	2020/8/6	-1602bp/-1602bp	N2F1	survival
17/39,		205	♂	2020/8/26	2020/8/6	wt/wt	N2F1	survival
17/39,		206	♂	2020/8/26	2020/8/6	wt/wt	N2F1	survival
17/39,		207	♂	2020/8/26	2020/8/6	-1602bp/wt	N2F1	survival
17/39,		208	♀	2020/8/26	2020/8/6	wt/wt	N2F1	survival
17/39,		209	♀	2020/8/26	2020/8/6	-1602bp/-1602bp	N2F1	survival
28/43,	-1602bp/wt	210	♂	2020/8/27	2020/8/7	wt/wt	N2F1	survival
28/43,		211	♂	2020/8/27	2020/8/7	-1602bp/wt	N2F1	survival
28/43,		212	♀	2020/8/27	2020/8/7	-1602bp/wt	N2F1	survival
28/43,		213	♀	2020/8/27	2020/8/7	-1602bp/-1602bp	N2F1	survival
28/43,		214	♀	2020/8/27	2020/8/7	-1602bp/wt	N2F1	survival

Table S3. Information on antibodies used in this study

Antigen	Reactivity	Company	Catalog #	Dilution			
				WB	IHC	IF	ChIP
**CEBPδ**	Human, Mouse	GeneTex	GTX115047	1:1000	1:100	1:50	1:50
**COX-2**	Human, Mouse	CST	12282S	1:2000	1:100		
**11β-HSD1**	Human	Abcam	ab157223	1:1000			
**11β-HSD1**	Mouse	Abcam	ab39364	1:2000	1:100		
**GAPDH**	Human, Mouse	Proteintech	60004-1	1:10000			
**Vimentin**	Human	Santa Cruz	sc-6260	1:10000		1:200	
**Lamin A/C**	Human	CST	4777S	1:1000			
**E-cadherin**	Human	CST	3195S	1:1000			
**β-actin**	Human	Proteintech	66009-1	1:10000			

WB, Western blotting; IHC, immunohistochemical staining; IF, immunofluorescence staining; ChIP, chromatin immunoprecipitation assay.

Table S4. Primer sequences used in this study.

	Gene	Forward Primer (5’-3’)	Reverse primer (5’-3’)
qRT-PCR	CEBPD	CATCGACTTCAGCGCCTACA	TTGAAGAGGTCGGCGAAGAG
	HSD11B1	GCAGCCTCAGCACACTACAT	CATGTCTAGTCCTCCCATGAGC
	PTGS2	TGTGCAACACTTGAGTGGCT	ACTTTCTGTACTGCGGGTG
	GAPDH	CCCCTCTGCTGATGCCCCCA	TGACCTTGGCCAGGGGTGCT
	CEBPA	TCGGTGGACAAGAACAGCAA	TTGTCACTGGTCAGCTCCAG
	CEBPB	AAGCACAGCGACGAGTACAA	ACAGCTGCTCCACCTTCTTC
	CEBPG	GGCTTACAGCAGGTTCCTCA	ATGTTGTTCCTCTCTCGGCG
	CEBPE	CAATCCCCTGCAGTACCAAGT	CCAAAGGGGCCTTGAGAACG
	DDIT3	CCTGGAAATGAAGAGGAAGAATC	CTGACTGGAATCTGGAGAGTGA
ChIP	HSD11B1	CAGTCCTGTACAGTCATGAGCTTG	GTGCTAGCCAATTTCCCTGTCA
	PTGS2	AGCTTCCTGGGTTTCCGATT	GCCCATGTGACGAAATGACTG

qRT-PCR, quantitative real time PCR; ChIP, chromatin immunoprecipitation assay.
